# A novel “prime and pull” strategy mediated by the combination of two dendritic cell-targeting designs induced protective lung tissue-resident memory T cells against H1N1 influenza virus challenge

**DOI:** 10.1186/s12951-023-02229-y

**Published:** 2023-12-13

**Authors:** Zhannan Wang, Yingkai He, Wenfeng Wang, Yawen Tian, Chongbo Ge, Futing Jia, Tongyu Zhang, Gerui Zhang, Mingyue Wang, Jinshuo Gong, Haibin Huang, Jianzhong Wang, Chunwei Shi, Wentao Yang, Xin Cao, Yan Zeng, Nan Wang, Aidong Qian, Yanlong Jiang, Guilian Yang, Chunfeng Wang

**Affiliations:** College of Veterinary Medicine, Jilin Provincial Engineering Research Center of Animal Probiotics, Jilin Provincial Key Laboratory of Animal Microecology and Healthy Breeding, Engineering Research Center of Microecological Vaccines (Drugs) for Major Animal Diseases, Ministry of Education, Jilin Agricultural University, Changchun, 130118 China

**Keywords:** Influenza virus, Nanoparticle vaccine, DC targeting, Sequential immunization, T_RM_ cell

## Abstract

Vaccination is still the most promising strategy for combating influenza virus pandemics. However, the highly variable characteristics of influenza virus make it difficult to develop antibody-based universal vaccines, until now. Lung tissue-resident memory T cells (T_RM_), which actively survey tissues for signs of infection and react rapidly to eliminate infected cells without the need for a systemic immune reaction, have recently drawn increasing attention towards the development of a universal influenza vaccine. We previously designed a sequential immunization strategy based on orally administered *Salmonella* vectored vaccine candidates. To further improve our vaccine design, in this study, we used two different dendritic cell (DC)-targeting strategies, including a single chain variable fragment (scFv) targeting the surface marker DC-CD11c and DC targeting peptide 3 (DCpep3). Oral immunization with *Salmonella* harboring plasmid pYL230 (S230), which displayed scFv-CD11c on the bacterial surface, induced dramatic production of spleen effector memory T cells (T_EM_). On the other hand, intranasal boost immunization using purified DCpep3-decorated 3M2e-ferritin nanoparticles in mice orally immunized twice with S230 (S230inDC) significantly stimulated the differentiation of lung CD11b^+^ DCs, increased intracellular IL-17 production in lung CD4^+^ T cells and elevated chemokine production in lung sections, such as CXCL13 and CXCL15, as determined by RNAseq and qRT‒PCR assays, resulting in significantly increased percentages of lung T_RM_s, which could provide efficient protection against influenza virus challenge. The dual DC targeting strategy, together with the sequential immunization approach described in this study, provides us with a novel “prime and pull” strategy for addressing the production of protective T_RM_ cells in vaccine design.

**Supplementary Information:**

The online version contains supplementary material available at 10.1186/s12951-023-02229-y.

## Introduction

The consistent circulation of influenza virus worldwide still causes great economic loss and public threats to humans. A series of subtypes, such as H7N9 [1], H5N1 [2], H5N6 [3], H1N1 [4] and H3N8 [5], have been reported to pose severe threats to public health in recent years. Among these subtypes, the reemergence of H1N1 influenza virus between 2021 and 2023 [6] had dramatic effects on our society, especially considering its somewhat similar clinical symptoms compared with those of SARS-CoV-2, resulting in increased concerns about influenza viruses. Although vaccination remains the most effective method for preventing influenza-related illness, seasonal influenza vaccine effectiveness ranges from 10 to 60% due to vaccine strains that may not be well matched to circulating strains [7]. Therefore, the development of a universal influenza vaccine has been highly desirable in recent years. Some well-known protective antigens, such as the ectodomain of matrix protein 2 (M2e) [8, 9], the headless hemagglutinin (HA) stalk region (HA2) [10], the conserved nucleoprotein, matrix protein [11] and neuraminidase [12], have been included in previous studies. To further improve vaccine design, some novel technologies, such as mRNA vaccines [11], nanoparticle vaccines [8, 13], lymph node follicle targeting [9] and dendritic cell (DC) targeting strategies [14], have recently been drawing great attention, especially after the outbreak of SARS-CoV-2 in 2020. On the other hand, CD8 T cell responses against invariable components of the virus provide protection against different strains. Vaccines that induce this type of protective immunity would provide major health and economic benefits [15]. In particular, the ability of memory CD8 T cells to proliferate and gain multifunctional cytotoxic function quickly upon the detection of viruses, which ensures the immediate control of reinfection [16], has drawn increasing attention recently. Among the memory T cells, tissue-resident memory T (T_RM_) cells with long-lived and self-sustaining properties have been identified in skin and mucosal surfaces, including the lung [15], displaying a unique molecular signature that distinguishes them from the circulating memory T-cell pool and results in optimal survival and function within their local environment [17]. T_RM_s are typically defined by the cell markers CD69 and CD103, while CD69 is an early marker of T-cell activation that interferes with sphingosine-1-phosphate receptor function to facilitate T-cell retention and local memory formation [18], CD103 binds E-cadherin on epithelial cells to assist tissue retention [19]. Notably, it has been demonstrated that the presence of influenza-specific CD4 and CD8 T_RM_ cells within the lung could provide effective protection against virus infection [20–22]. Therefore, a strategy that could efficiently induce the generation of lung T_RM_s should be the future direction for influenza vaccine design.

Antigen-presenting cells (APCs), especially DCs, play a critical role in the interaction between innate immunity and adaptive immunity after infection or immunization. Targeting antigens toward molecules or chemokine receptors on APCs, such as CD11c [23], DEC-205 [24], Xcr1 [25], major histocompatibility complex class II (MHC-II) [26] and macrophage galactose-type lectin (MGL) [27], could dramatically stimulate enhanced immunogenicity. To date, monoclonal antibodies [28], single chain variable fragments (scFvs) [23], nanobodies [29] and DC-targeting peptides (DCpep) [30] have been employed in the design of DC-targeting vaccines. For example, we constructed a novel *Lactobacillus plantarum* strain with surface displayed scFv targeting the CD11c marker of mouse DCs (scFv-CD11c) [31] previously using a truncated poly-γ-glutamic acid synthetase A (pgsA’) anchoring sequence [32], the surface anchored scFv-CD11c significantly increased the cellular uptake of bacteria in mouse BMDCs [33] and stimulated elevated humoral immune responses against influenza virus H1N1 strain using an HA coexpressed model antigen, especially the cellular immune response [23]. On the other hand, DCpep is a series of short peptides that can mediate the specific interaction between DCpep-fused antigen and DCs in swine [34], mice [35] or chickens [36], resulting in increased specific humoral and cellular immune responses. Notably, the smaller size makes DCpep easier to manipulate than other DC-targeting molecules. Among the different DCpeps that have been reported, DCpep3 (SLSLLTMPGNAS) has been shown to be the most effective option in mice immunized with porcine circovirus type 2 Cap-DCpep3 virus-like particles (VLPs), which exhibited significantly higher levels of Cap-specific antibodies, neutralizing antibodies and intracellular cytokines than those with other DCpeps integrated or wild-type Cap VLPs without any DCpep [37]. In addition to the enhanced conventional humoral immune response, it has been demonstrated that antibody-targeted vaccination of lung DCs generates CD8^+^ T_RM_ cells that are highly protective against influenza virus infection, in which the recognition of antigens presented locally by DCs and transforming growth factor-β (TGFβ) signaling are both needed [15].

In our previous study [38], we designed a *Salmonella*-mediated in vivo nanoparticle influenza vaccine based on the 24-mer self-assembled ferritin nanocage and M2e domain of H1N1 virus. The sequential immunization using purified nanoparticles by intranasal boost after the oral administration of the *Salmonella* strain significantly increased the cellular immune response and elevated the levels of effector memory T (T_EM_) cells and T_RM_ cells in the lungs, resulting in improved protection against low-dose virus challenge by H1N1. Since DC-targeting vaccines have been demonstrated to specifically increase the generation of protective lung T_RM_ cells [15], we aimed to determine whether the combination of two DC-targeting designs and *Salmonella-*mediated sequential immunization could further elevate the production of lung T_RM_ cells to yield better protection against influenza challenge. In this study, we applied the surface-displayed scFv-CD11c and DC-targeting peptide strategies to orally administered *Salmonella* and intranasal boost-immunized nanoparticles, respectively. The surface-displayed scFv-CD11c on the *Salmonella* strain dramatically stimulated the production of T_EM_ cells in the circulation after oral administration, whereas the intranasal boost with purified 3M2e-ferritin nanoparticles decorated with DCpep3 significantly increased the population of lung CD4 and CD8 T_RM_ cells, resulting in improved protection against H1N1 challenge.

## Results

### Surface-displayed scFv-CD11c dramatically increased the cellular uptake of *Salmonella* in mouse bone marrow-derived cells (BMDCs)

**Fig. 1:**
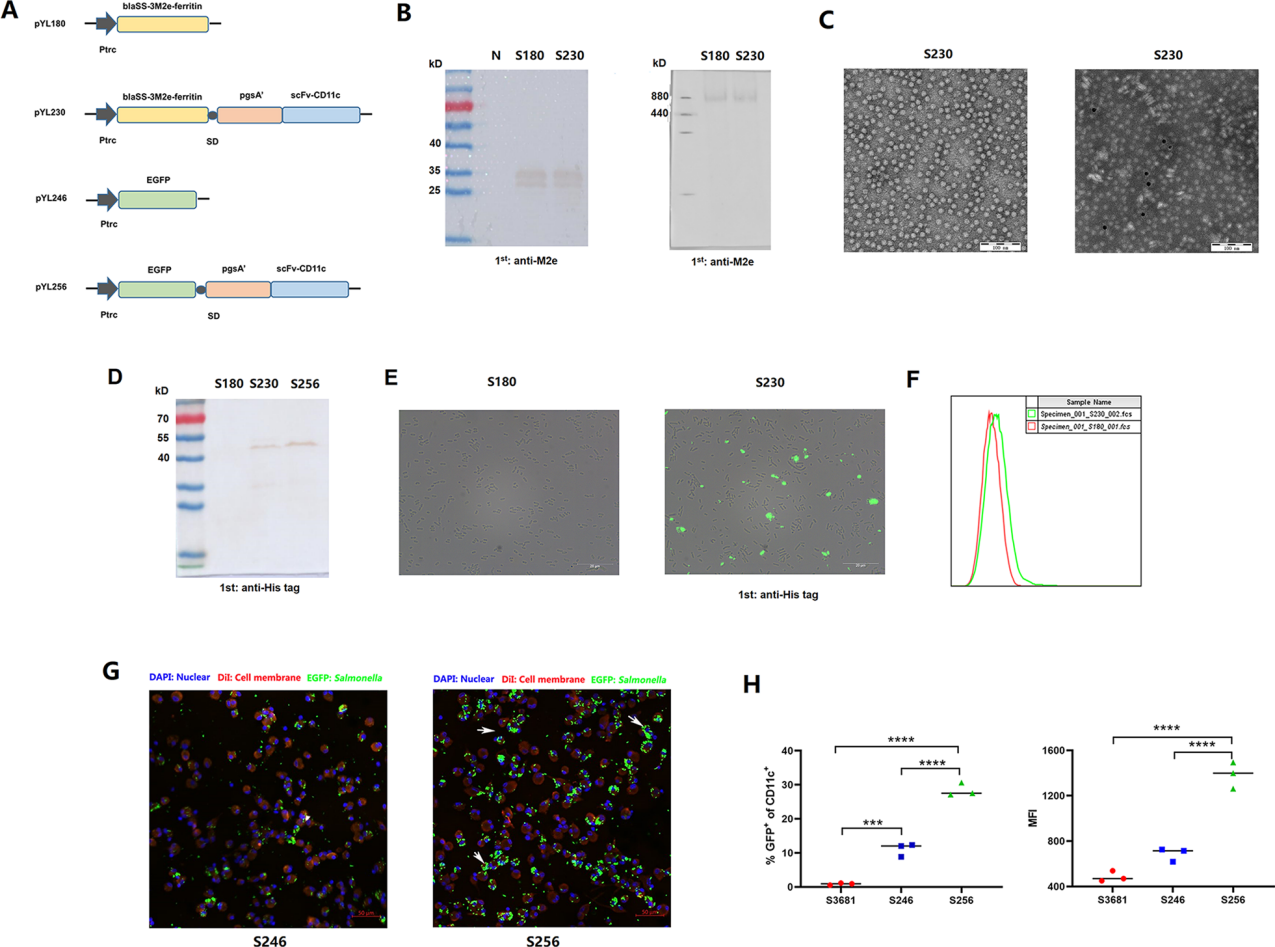
Plasmid construction, protein synthesis and characteristics of the single chain variable fragment of the anti-CD11c (scFv-CD11c) antibody. Plasmid pYL180, encoding three copies of M2e (H1N1) (3M2e), and pYL246, encoding an EGFP fluorescent protein, were used as parental plasmids. Then, a Shine‒Dalgarno (SD) sequence together with the pgsA’ anchoring sequence and scFv-CD11c sequence was inserted into the SacII site, yielding pYL230 and pYL256, respectively (**A**). Synthesis of 3M2e in *Salmonella* harboring plasmids pYL180 and pYL230, named S180 and S230, respectively, was confirmed by reducing (left) and non-reducing (right) western blot using an M2e-specific antibody as the primary antibody (**B**). Purified nanoparticles from S180 and S230 were subjected to a transmission electron microscopy (TEM) assay (left) and immune electron microscopy (right) using an M2e-specific antibody as the primary antibody (**C**).The production of surface-displayed scFv-CD11c in S230 and *Salmonella* containing pYL256 (S256) was also determined using a His-tag antibody by western blot (**D**). Surface-displayed scFv-CD11c on S230 was evaluated by immunofluorescence analysis using His-tag primary antibody and FITC-labeled secondary antibody, S180 was also included as a mock control (**E**). The presence of scFv-CD11c on the surface of S230 was also confirmed by flow cytometry (**F**). Mouse bone marrow-derived cells (BMDCs) were incubated with S246 and S256 for 2 h, and then the cells were detected by microscopy. Blue, DAPI-labeled nuclei; red, DiI-labeled cell membrane; green, *Salmonella* producing EGFP (**G**). The cellular uptake of *Salmonella* S246 and S256 was also determined by flow cytometry. The percentages of CD11c^+^EGFP^+^ BMDCs as well as the mean fluorescence intensity were calculated (**H**) (n = 3, ***P < 0.001, ****P < 0.0001)

The construction of plasmid pYL180 harboring three copies of M2e (H1N1) (3M2e) fused with ferritin 24-mer nanoparticles was described previously [38]. Then, a scFv-CD11c fragment designed for surface-displaying DCs targeting a scFv antibody on bacteria [31] was inserted after the 3M2e-ferritin operon by a Shine-Dalgarno (SD) sequence [39] to yield pYL230 (Fig. 1A). To demonstrate the effects of scFv-CD11c on *Salmonella*, plasmids that synthesized EGFP fluorescence alone (pYL246) or together with the scFv-CD11c fragment (pYL256) were also constructed using the same strategy (Fig. 1A). The successfully produced M2e components were then confirmed by both reducing and non-reducing western blot assays using the M2e-specific antibody (Fig. 1B). To further confirm the presence of 3M2e-ferritin nanoparticles in *Salmonella*, proteins were extracted as described previously [38], and then transmission electron microscopy (TEM) and immunoelectron microscopy were performed using the M2e-specific antibody. The results showed that M2e could be displayed successfully on the surface of 24-mer nanoparticles, as expected (Fig. 1C).

The surface-displayed pgsA’-scFvCD11c was evaluated by a His-tag-specific western blot using the extraction of cell wall components from *Salmonella* harboring both pYL180 and pYL230, named S180 and S230, respectively (Fig. 1D). In addition, an immunofluorescence assay was also conducted using the His tag antibody as the primary antibody, and the results indicated that scFv-CD11c could be detected on the surface of S230 but not S180 using a FITC-labeled secondary antibody (Fig. 1E). An obvious fluorescence signal shift could also be observed in S230 strains compared with S180 by flow cytometry analysis (Fig. 1F), indicating the successful displayed scFv-CD11c on the surface of S230. Then, the EGFP-synthesized *Salmonella* strains harboring pYL246 and pYL256, named S246 and S256, respectively, with or without surface-displaying scFv-CD11c, were incubated with BMDCs in vitro for 2 h. The results demonstrated that the presence of scFv-CD11c on *Salmonella* could dramatically increase the cellular uptake of green fluorescence in the S256 group (Fig. 1G). The cellular uptake of EGFP signals was also determined by flow cytometry, and the results showed that significantly increased fluorescence-positive cells (*P* < 0.001), as well as the mean fluorescence intensity (MFI) (*P* < 0.001) (Fig. 1H), could be detected in S256-treated BMDCs compared with both S3681 empty vector control and S246 strains. All the results mentioned above indicated that the surface-displayed scFv-CD11c on *Salmonella* could efficiently increase the cellular uptake of bacteria by BMDCs.

### DCs targeting *Salmonella* could moderately stimulate the maturation of DCs in vitro and in vivo

**Fig. 2:**
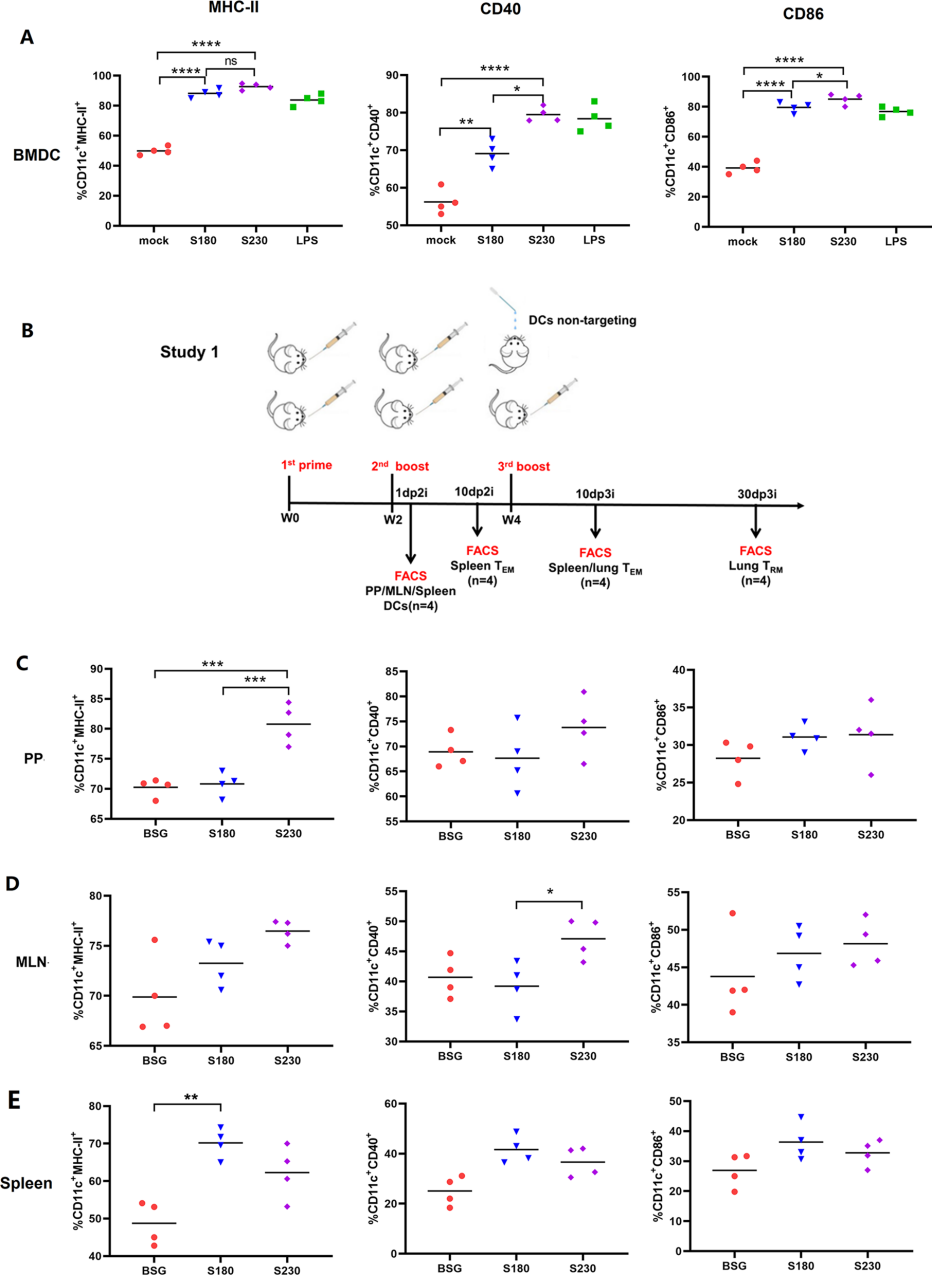
Dendritic cells (DCs) targeting *Salmonella* stimulated DC differentiation in vitro and in vivo. The BMDCs were prepared and incubated with *Salmonella* strains χ11802 (pYL180) and χ11802 (pYL230), named S180 and S230, respectively, with a multiple of infection (MOI) of 100 for 2 h. BSG and LPS (0.5 μg/mL) were also included as mock and positive controls. The maturation markers MHC-II, CD40 and CD86 were determined by flow cytometry (**A**). An illustration of the animal experiment (Study 1) is shown (**B**). One day after the second immunization, PPs (**C**), MLNs (**D**) and spleens (**E**) were collected and analyzed by flow cytometry to evaluate the expression levels of MHC-II, CD40 and CD86 (n = 4, *P < 0.05, **P < 0.01, ***P < 0.001, ****P < 0.0001, ns, not significant)

BMDCs were isolated and incubated with S180 or S230 as described below, and maturation was determined by flow cytometry. The results showed that both S180 and S230 could dramatically stimulate the activation of BMDCs, in which S230 could significantly increase the percentages of CD11c^+^CD40^+^ and CD11c^+^CD86^+^ BMDCs compared with those of S180 (Fig. 2A), indicating that the surface-displayed scFv-CD11c could benefit the maturation of DCs in vitro. Then, an animal study was designed as indicated (Fig. 2B), and the maturation of DCs in vivo was also measured using single-cell lymphocytes collected from Peyer’s patches (PPs) (Fig. 2C), mesenteric lymph nodes (MLNs) (Fig. 2D) and spleens (Fig. 2E) one day after the 2nd immunization with S180 and S230. In most cases, oral treatment with S230 did not result in the expected elevation of cell surface markers for DC maturation compared with S180, although there was a significantly increased percentage of CD11c^+^MHCII^+^ cells in PPs (P < 0.001) (Fig. 2C).

### Surface-displayed scFv-CD11c induced increased generation of T_EM_ cells in the spleen after oral administration

**Fig. 3:**
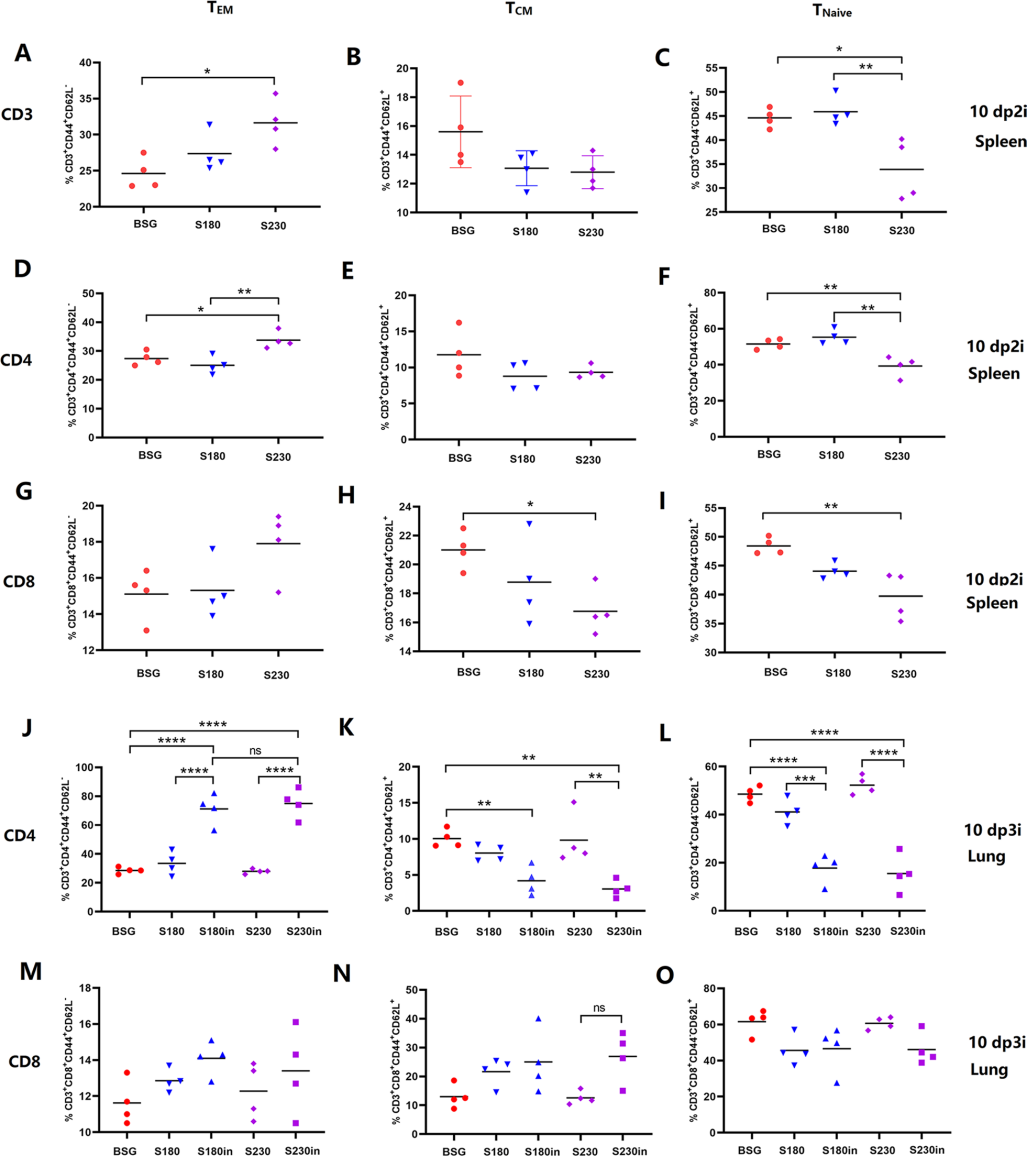
Production of effector memory T cells (T_EM_) before and after intranasal boost immunization in Study 1. At 10 days post-2nd immunization (10 dp2i), spleen samples were collected and subjected to flow cytometry analysis to determine the percentages of CD44^+^CD62L^−^ T_EM_ cells, CD44^+^CD62L^+^ T_CM_ cells and CD44^−^ CD62L^+^ T_Naive_ cells in the CD3 (**A**–**C**), CD4 (**D**–**F**) and CD8 (**G**–**I**) subgroups. At 10 days post-3rd immunization (10 dp3i), lung samples were collected and subjected to flow cytometry analysis to determine the percentages of T_EM_, T_CM_ and T_Naive_ cells in the CD4 (**J**–**L**) and CD8 (**M**–**O**) subgroups (n = 4, *P < 0.05, **P < 0.01, ***P < 0.001, ****P < 0.0001, ns, not significant). Notably, *Salmonella* strain χ11802 harboring plasmids pYL180 and pYL230 was designated S180 and S230, respectively

Spleen samples were collected at 10 days post-2nd immunization (10 dp2i), and the percentages of T_EM_ (CD44^+^CD62L^−^), T_CM_ (CD44^+^CD62L^+^) and T_Naive_ (CD44^−^CD62L^+^) cells among CD3 (Fig. 3A–C), CD4 (Fig. 3D–F) and CD8 (Fig. 3G–I) T cells were determined by flow cytometry. In general, the oral administration of S230, which could synthesize and display scFv-CD11c on the surface of the *Salmonella* strain by the pgsA’ anchoring sequence, tended to increase the percentage of CD3 T_EM_ cells compared with that in after S180 administration (without scFv-CD11c construction) (Fig. 3A). Meanwhile, the percentages of both T_CM_ (Fig. 3B) and T_Naive_ (Fig. 3C) cells appeared to decrease in the mice immunized with DCs targeting *Salmonella*. In detail, both CD4 (Fig. 3D) and CD8 (Fig. 3G) T_EM_ cells in S230 immunized mice appeared to increase compared with those in either buffered saline with gelatin (BSG) [40] or S180, especially for the CD4 T_EM_ cells. Notably, similar trends regarding the percentages of CD4 T_CM_ (Fig. 3E), CD4 T_Naive_ (Fig. 3F), CD8 T_CM_ (Fig. 3H) and CD8 T_Naive_ (Fig. 3I) were observed compared to CD3 T_CM_ (Fig. 3B) and CD3T_Naive_ (Fig. 3C) subtypes.

### An intranasal boost with purified nanoparticles induced a similar population of T_EM_ cells in the lungs of S180- and S230-primed mice

Mice were immunized twice by oral administration of S180 or S230, followed by a third intranasal boost immunization using purified nanoparticles, named S180in and S230in, respectively. The generation of T_EM_ cells in lung sections was determined by flow cytometry at 10 days post 3rd immunization (dp3i), and the results showed that three oral administrations of S180 or S230 did not induce any generation of CD4^+^ T_EM_ (Fig. 3J) or CD8^+^ T_EM_ (Fig. 3M) cells in the lungs, whereas intranasal boost immunization in both the S180in and S230in groups significantly stimulated the production of lung T_EM_ cells, especially CD4^+^ T_EM_ (*P* < 0.001) (Fig. 3J). Notably, no significant difference could be observed in S180in and S230in mice regarding the percentages of T_EM_ cells (*P* > 0.05) (Fig. 3J), indicating that the DCs targeting *Salmonella* did not increase the production of lung T_EM_ cells compared with that of nontargeting *Salmonella*. Not surprisingly, similar percentages of CD4^+^ (Fig. 3K, L) and CD8^+^ (Fig. 3N, O) T_CM_ and T_Naive_ cells in the S180in and S230in groups were also observed, which were significantly decreased compared with those in either the BSG or S180 and S230 groups, respectively. Notably, the generation of lung CD8^+^ T_EM_, T_CM_ and T_Naive_ cells was not severely affected by oral or intranasal immunization, even though a similar trend was observed compared with that of CD4^+^ T cells (Fig. 3M–O). In addition, the percentages of both CD4^+^ and CD8^+^ T_EM_ cells in spleens were also determined at 10 dp3i, and the results showed that there were no significant differences among all the groups (data not shown). The results regarding the production of T_EM_ cells showed that orally administered *Salmonella* with surface-displayed scFv-CD11c could efficiently stimulate the generation of spleen T_EM_ cells but not lung T_EM_ cells. On the other hand, intranasal boost immunization using purified nanoparticles could significantly increase the percentages of lung T_EM_ cells in mice primed with either DCs targeting or non-targeting *Salmonella*, with a very similar level, indicating that the increased lung T_EM_ cells were caused by intranasal boost immunization with nanoparticles.

### Intranasal boost immunization elicited more expansion of both CD4^+^ and CD8^+^ lung T_RM_ cells in S230-primed mice than in S180-primed mice

**Fig. 4:**
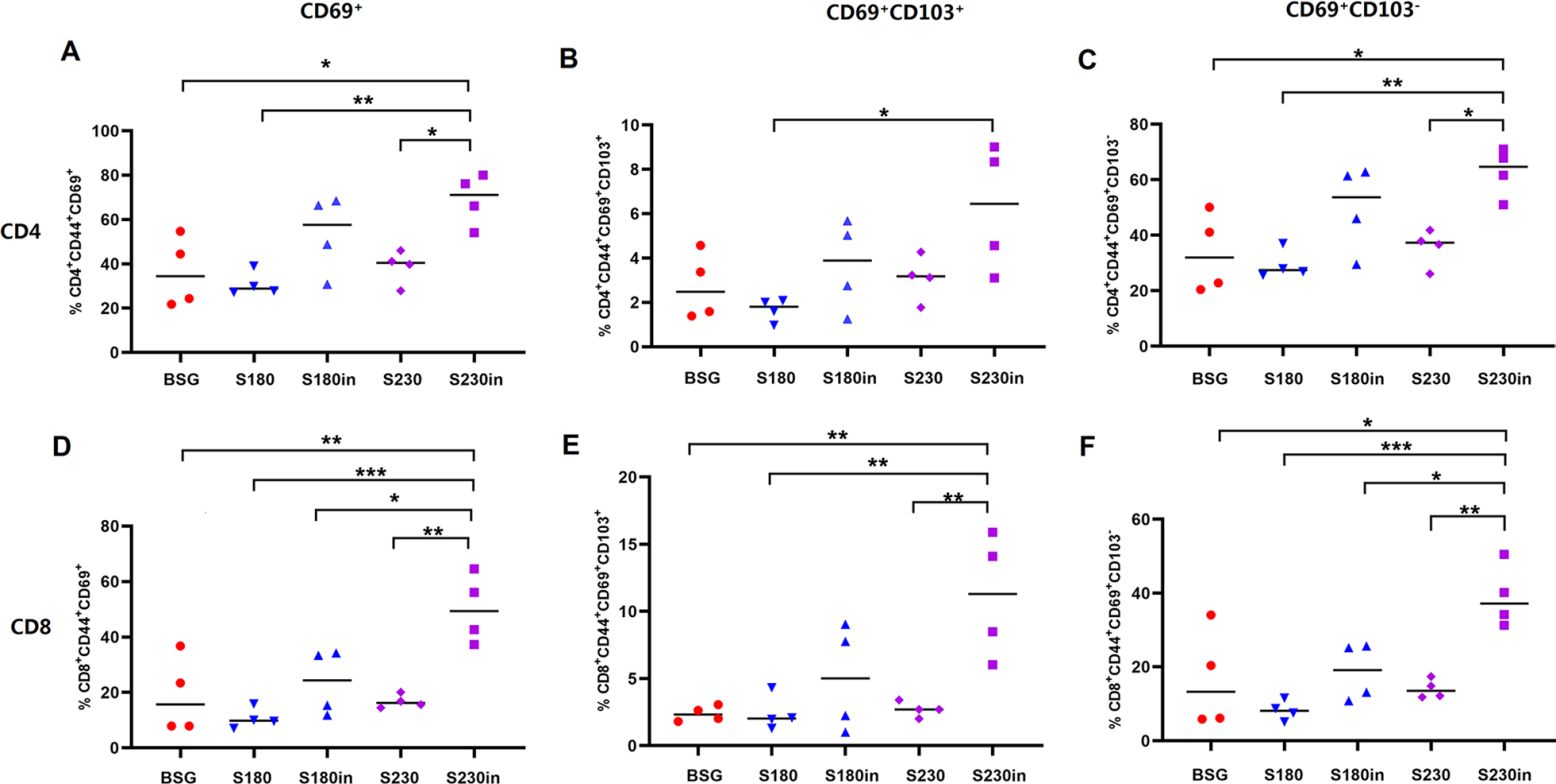
Generation of CD4^+^ and CD8^+^ lung-resident memory T (T_RM_) cells in lung tissues. At 30 days post-3rd immunization in Study 1, single-cell suspensions from the lung samples were collected and subjected to FACS to determine the percentages of CD4^+^CD44^+^CD69^+^ (**A**), CD4^+^ CD44^+^CD69^+^CD103^+^ (**B**), CD4^+^ CD44^+^CD69^+^CD103^−^ (**C**), CD8^+^CD44^+^CD69^+^ (**D**), CD4^+^ CD44^+^CD69^+^CD103^+^ (**E**) and CD8^+^ CD44^+^CD69^+^CD103^−^ (**F**) T_RM_ cells (n = 4, *P < 0.05, **P < 0.01, ***P < 0.001). Notably, *Salmonella* strain χ11802 harboring plasmids pYL180 and pYL230 was designated S180 and S230, respectively. Two oral immunizations with S180 and S230 followed by the 3rd intranasal boost immunization with purified nanoparticles were named S180in and S230in, respectively

The lung samples were collected at 30 dp3i and analyzed by flow cytometry to determine the presence of CD69^+^ and/or CD69^+^CD103^+^ T_RM_ cells. As expected, three oral immunizations with S180 or S230 did not result in obvious generation of T_RM_ cells (Fig. 4A). On the other hand, the 3rd intranasal boost immunization with purified nanoparticles in both S180- and S230-primed mice dramatically increased the production of CD4^+^ (Fig. 4A–C) and CD8^+^ (Fig. 4D–F) T_RM_ cells. In particular, intranasal boost immunization significantly increased the percentages of both CD4^+^ (Fig. 4A) and CD8^+^ (Fig. 4D) lung T_RM_ cells in S230-primed mice compared those with S180-primed mice. In detail, both CD69^+^CD103^+^ T_RM_ cells (Fig. 4B, E) and CD69^+^CD103^−^ T_RM_ cells (Fig. 4C, F) were significantly increased after the 3rd intranasal boost immunization in S230-primed mice. In fact, the presence of T_RM_ cells in mouse lungs was also determined at 6 months after the last immunization, and the results demonstrated that the persistence of lung T_RM_ cells lasted for at least half a year (data not shown). Since the percentages of lung T_EM_ cells in the S230in and S180in groups at 10 dp3i were similar (Fig. 3J, M), the evidently increased generation of lung T_RM_ cells in the S230in group (Fig. 4A, D) was most likely not from in situ T_EM_ cells but instead from the increased circulating T_EM_ cell pool (Fig. 3A, D and G). To further confirm this hypothesis, FTY720 [41] was used to prevent trafficking of peripheral memory T cells into the lungs before intranasal boost immunization. The results showed that there was no obvious generation of either CD4^+^ (Additional file 1: Fig. S1A–C) or CD8^+^ (Additional file 1: Fig. S1D–F) lung T_RM_ cells in any of the three test groups, indicating that the observed production of lung T_RM_ cells was most likely from the circulating T-cell pool instead of in situ generation.

### Intranasal boost immunization with DCs targeting nanoparticles stimulated the activation of DCs in vivo

**Fig. 5:**
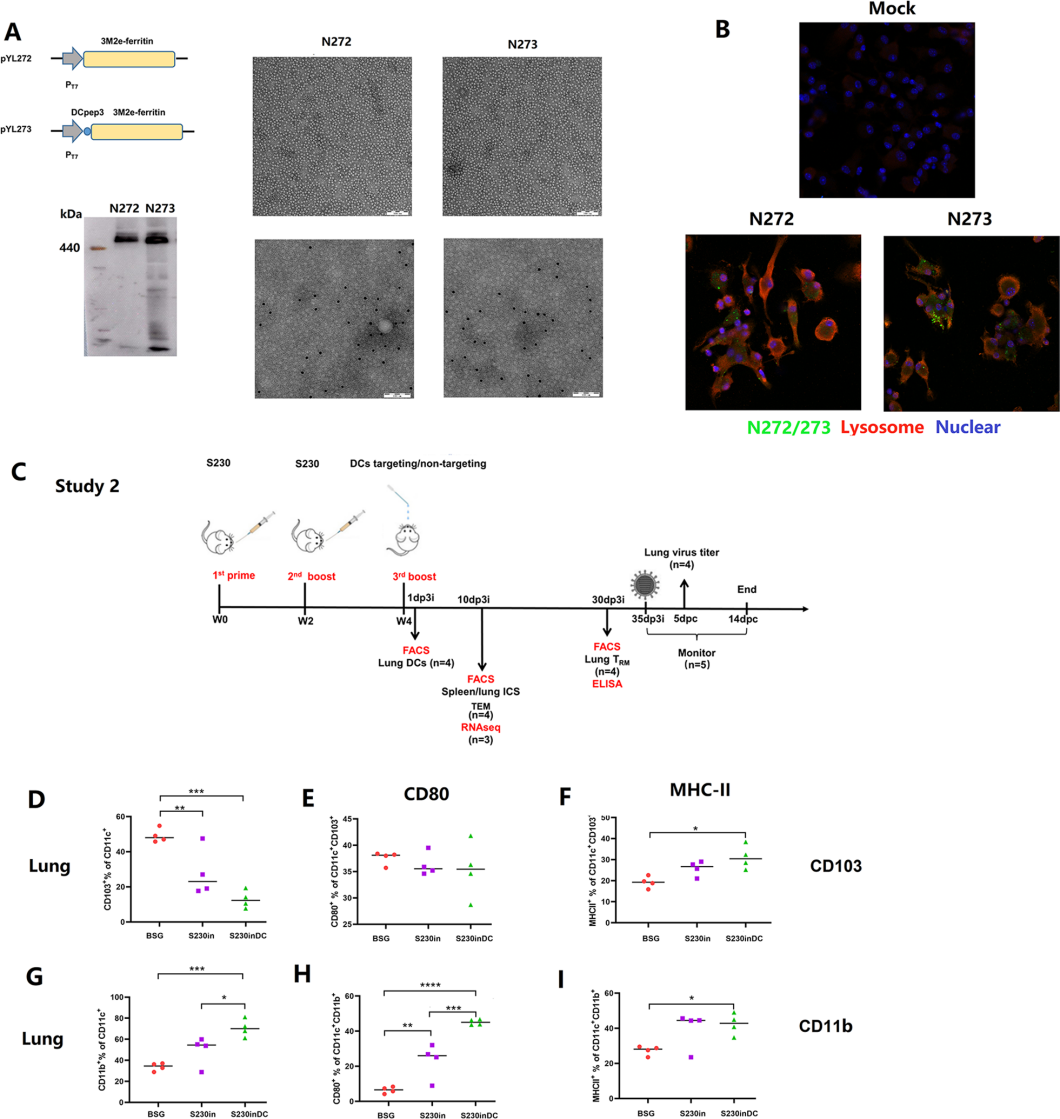
Intranasal boost immunization with DCs targeting nanoparticles stimulated the maturation of CD11b^+^ DCs in the lungs of mice. Plasmids pYL172 and pYL273, which synthesize 3M2e-ferritin or DCpep3-fused 3M2e-ferritin, were constructed, and the expression of M2e was confirmed by non-reducing western blotting, transmission electron microscopy (TEM) and immune electron microscopy (**A**). The purified DCs targeting/non-targeting nanoparticles from pYL273/pYL272, named N273/N272, were labeled with Flamma 496 NHS (green) and incubated with BMDCs for 16 h. After that, cellular lysosomes and nuclei were stained with LAMP1 antibody (red) and DAPI (blue), respectively (**B**). An illustration of the animal experiment (Study 2) is shown (**C**) in which mice were orally immunized with S230 twice, followed by an additional 3rd intranasal boost immunization with N272 or N273. One day after the 3rd intranasal boost immunization, lung DCs were analyzed by flow cytometry to determine the percentages and activation of CD103^+^ (**D**–**F**) and CD11b^+^ DCs (**G**–**I**). (n = 4, *P < 0.05, **P < 0.01, ***P < 0.001, ****P < 0.0001). Notably, two oral immunizations with S230 followed by the 3rd intranasal boost immunization with either N272 or N273 were named S230in and S230inDC, respectively

DCpep3 was fused at the N-terminus of the 3M2e-ferritin fragment and inserted into the prokaryotic expression vector pET28a to generate plasmid pYL273, which could produce DC-targeting nanoparticles. For comparison purposes, a similar plasmid named pYL272 expressing non-DCs targeting 3M2e-ferritin nanoparticles was also constructed (Fig. 5A). After purification as described below, the presence of nanoparticles was confirmed by TEM, and the surface-located 3M2e and DCpep3-3M2e were confirmed by immunological TEM. The formation of 24-mer nanoparticles was also evaluated in a nonreducing western blot analysis in which a protein band of approximately 800 kDa could be observed (Fig. 5A).

To determine whether the presence of DCpep3 could enhance the cellular uptake of nanoparticles in BMDCs, purified nanoparticles from *E. coli* strain BL21 harboring pYL272 and pYL273 were incubated with BMDCs for 16 h. The results of the immunofluorescence experiment showed that DC-targeting nanoparticles could be absorbed into BMDCs more efficiently than non-DC-targeting nanoparticles (Fig. 5B). To further evaluate the effects of DC-targeting nanoparticles on lung DCs in vivo after intranasal boost immunization, a mouse study was performed as indicated (Fig. 5C), and DCs were collected at 24 h post immunization and analyzed by flow cytometry. The results demonstrated that the percentages of CD11c^+^CD103^+^ DCs were even decreased after immunization using DCs targeting nanoparticles (Fig. 5D), whereas the percentages of CD11c^+^CD11b^+^ DCs were significantly increased in DCpep3 nanoparticle-immunized mice compared those with either BSG- or non-DC-targeting nanoparticles (Fig. 5G), consistent with our previous results [38]. Both non-DC nanoparticles (Fig. 5E, F) and DCpep3 nanoparticles (Fig. 5H, I) could stimulate the maturation of lung DCs in vivo, especially DCpep3 nanoparticles, as shown by increased maturation levels of CD80 (Fig. 5H) and MHC-II (Fig. 5I).

### DCs targeting nanoparticles stimulated the bias of Th17 cells in the lung and significantly increased the polyfunctional CD8 T-cell response in the spleen

**Fig. 6:**
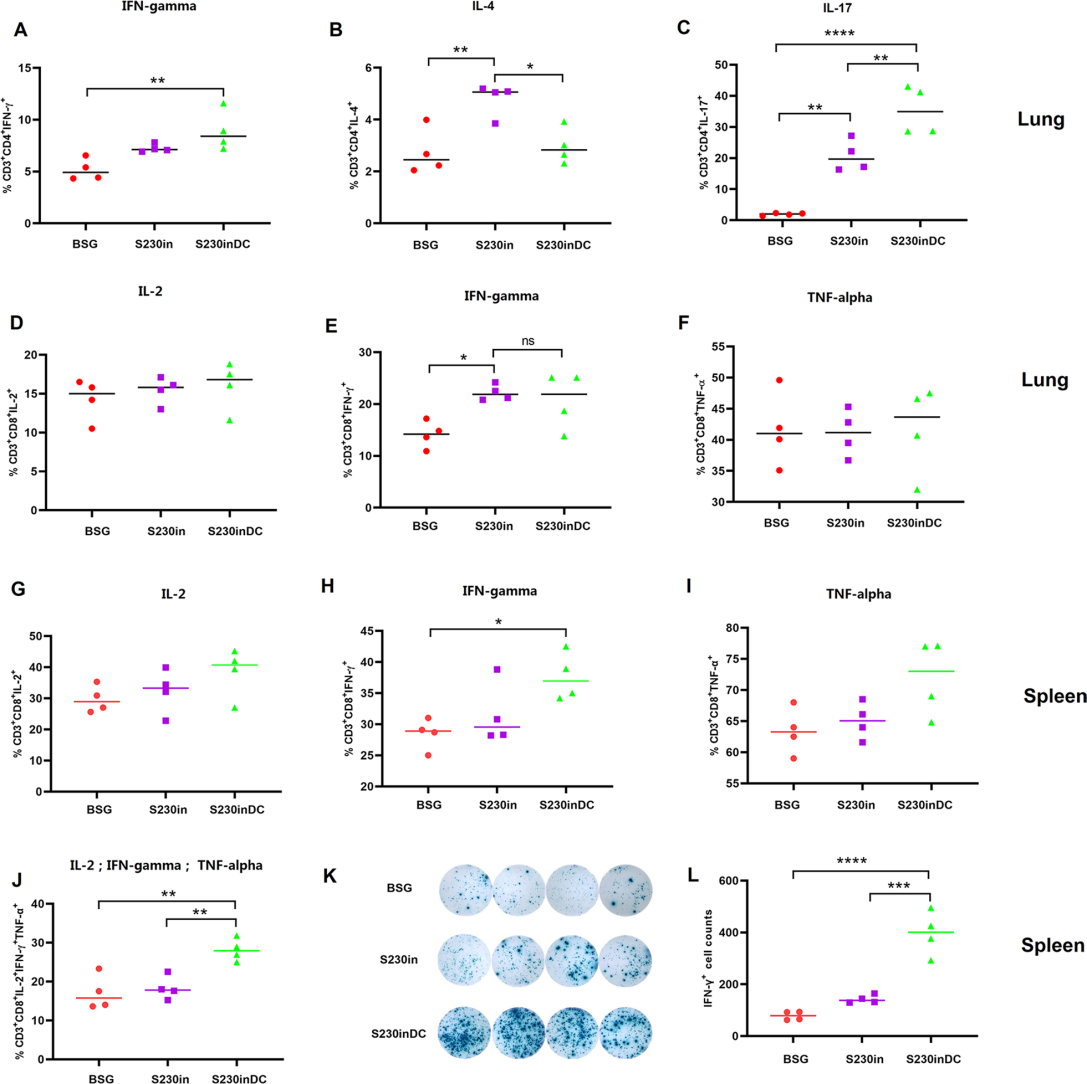
Intracellular cytokine production. At 10 days post-3rd immunization, the lung samples were subjected to flow cytometry analysis to evaluate the production of intracellular IFN-gamma (**A**), IL-4 (**B**), and IL-17 (**C**) in CD4 T cells, as well as IL-2 (**D**), IFN-gamma (**E**) and TNF-alpha (**F**) in CD8 T cells. The spleens were also collected, and the generation of IL-2 (**G**), IFN-gamma (**H**), TNF-alpha (**I**) and IL-2^+^ IFN-gamma^+^TNF-alpha^+^ (**J)** in CD8 T cells was measured. In addition, the intracellular production of IFN-gamma in the spleen was also measured by ELISpot assay (**K**, **L**)

The intracellular production of IFN-gamma, IL-4 and IL-17 in lung CD4^+^ T cells was determined as an indicator of Th1-, Th2- and Th17-biased immune responses. Similar levels of IFN-gamma were observed in the two intranasal immunization groups, whereas the DCs targeting nanoparticles stimulated a slightly higher level (Fig. 6A). Unexpectedly, intranasal immunization with non-targeting nanoparticles significantly increased the production of IL-4 in CD4^+^ T cells, whereas DCs targeting nanoparticles did not (Fig. 6B). In addition, intranasal immunization with non-DC-targeting nanoparticles could significantly elevate the intracellular IL-17 levels compared with those in the BSG control, whereas the DC-targeting design could efficiently stimulate the production of IL-17 further compared with that in either the BSG or the non-targeting S230inDC group (Fig. 6C), indicating that the DC-targeting strategy had evident effects on the Th17 subtype immune response. Then, the intracellular production of IL-2 (Fig. 6D), IFN-gamma (Fig. 6E) and TNF-alpha (Fig. 6F) in lung CD8^+^ T cells was also evaluated, and the results showed that only the IFN-gamma levels were increased compared with those in the BSG control (Fig. 6E), indicating that intranasal boost immunization had less effect on the production of intracellular cytokines in lung CD8^+^ T cells than in CD4^+^ T cells.

To further evaluate the cellular immune response induced by DCs targeting nanoparticles, the intracellular production of IL-2 (Fig. 6G), IFN-gamma (Fig. 6H) and TNF-alpha (Fig. 6I) in spleen CD8^+^ T cells was also measured. In general, the DC targeting design significantly stimulated the levels of all three cytokines compared with those in the other two groups. In particular, the IL-2^+^IFN-gamma^+^TNF-alpha^+^ polyfunctional CD8^+^ T cells were significantly increased in the DC-targeting boost group (Fig. 6J). An ELISpot assay was then performed to confirm the FACS results. As shown (Fig. 6K, L), the production of intracellular M2e-specific IFN-gamma cytokines in spleen lymphocytes was significantly increased compared with that in both BSG control and S230in lymphocytes. All the results described above indicated that intranasal boost immunization with DCs targeting nanoparticles had very different manners to affect host immune responses in the lung and spleen, which showed that intranasal immunization preferred to increase lung CD4^+^ Th17 cells as well as spleen CD8^+^ polyfunctional T cells and IFN-gamma secreting lymphocytes.

### RNAseq analysis of lung lymphocytes

**Fig. 7:**
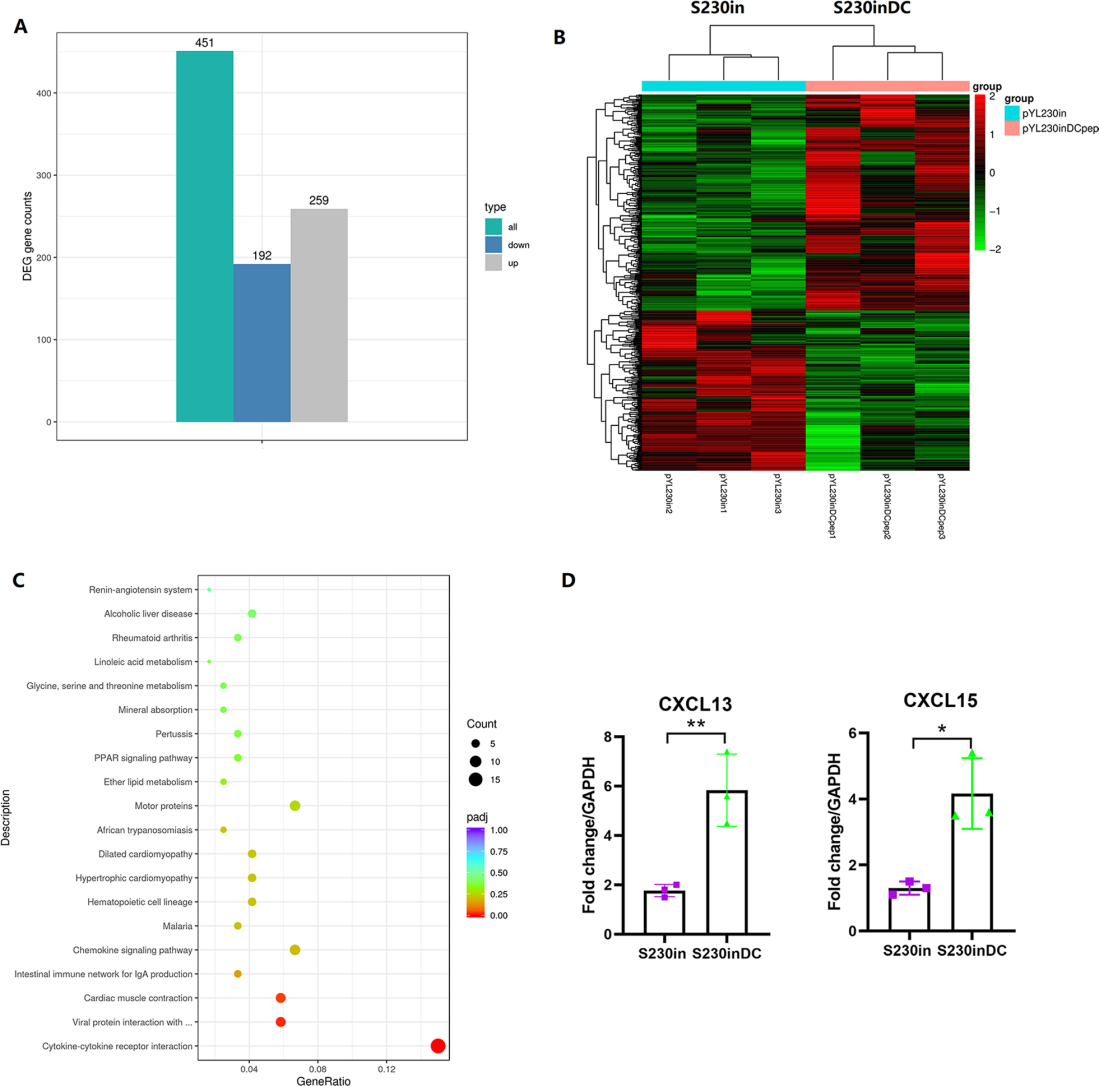
RNAseq analysis of lung lymphocytes. At 10 days post-3rd immunization, lymphocytes in lung sections were prepared and analyzed by RNAseq. The up/downregulated gene numbers (**A**), heatmap clustering (**B**) and KEGG enrichment analysis (**C**) were performed to compare the differences between mice immunized with S230in and S230inDC. qRT‒PCR was also performed to determine the RNA levels of the chemokines CXCL13 and CXCL15 in these groups (**D**)

Total RNA was extracted from lung lymphocytes at 10 dp3i, and the RNAseq analysis results showed that a total of 451 differentially expressed genes were identified, of which immunization with S230inDC resulted in 259 upregulated genes and 192 downregulated genes (Fig. 7A). The heatmap clustering showed that obviously different expression levels existed between S230in- and S230inDC-immunized mice (Fig. 7B). Subsequently, KEGG enrichment analysis indicated that terms were most significantly enriched in cytokine‒cytokine receptor interaction, viral protein interaction with cytokine and cytokine receptor, intestinal immune network for IgA production and chemokine signaling pathway (Fig. 7C), revealing that some enriched pathways might be related to interaction between chemokines and their receptors. Among the sequencing results, a number of chemokine genes, such as CXCL13 and CXCL15, were found to be dramatically upregulated in the S230inDC group compared with the S230 group. Therefore, CXCL13 and CXCL15 were selected to verify the results using qRT‒PCR, and the results confirmed that increased production of these two chemokines was also observed (Fig. 7D).

### Similar levels of CD4^+^ and CD8^+^ effector cells generated by DC-targeting and nontargeting nanoparticles in both the lung and spleen

**Fig. 8:**
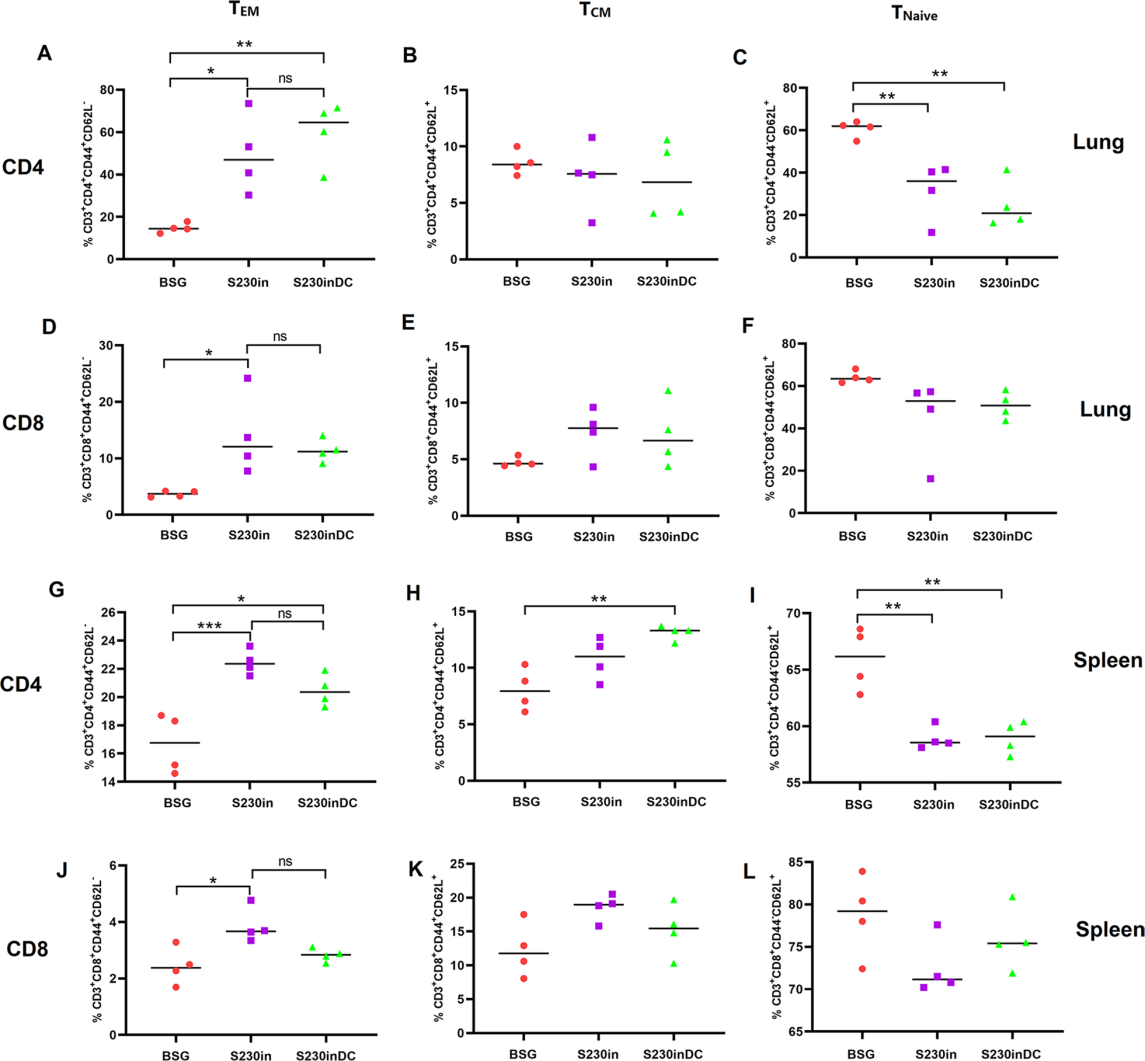
Production of effector memory T cells (T_EM_) after intranasal boost immunization in the lung and spleen in Study 2. At 10 days post-3rd immunization, lung and spleen samples were collected and subjected to flow cytometry analysis to determine the percentages of CD44^+^CD62L^−^ T_EM_ cells, CD44^+^CD62L^+^ T_CM_ cells and CD44^−^ CD62L^+^ T_Naive_ cells in the lung CD4 (**A**–**C**), lung CD8 (**D**–**F**), spleen CD4 (**G**–**I**) and spleen CD8 (**J**–**L**) subgroups. (n = 4, *P < 0.05, **P < 0.01, ***P < 0.001, ns: not significant)

We already showed that the intranasal boost with purified nontargeting nanoparticles in S230-primed mice could result in elevated production of lung T_EM_ cells (Fig. 3). Therefore, we determined whether intranasal immunization with DCs targeting nanoparticles could further increase the percentages of T_EM_ cells. The populations of T_EM_, T_CM_ and T_Naive_ cells regarding lung CD4 (Fig. 8A-C), lung CD8 (Fig. 8D–F), spleen CD4 (Fig. 8G–I) and spleen CD8 (Fig. 8J–L) were evaluated. The results demonstrated that intranasal immunization using both non-targeting and DC-targeting nanoparticles could significantly elevate the production of CD4^+^ (Fig. 8A, G) and CD8^+^ (Fig. 8D, J) T_EM_ cells in both the lung and spleen, however, there were no significant differences between these two types of nanoparticles, indicating that the DC targeting design did not further increase the production of T_EM_ cells. Altogether, with the previous observations of lung T_EM_ cells (Fig. 3), all the results demonstrated that intranasal immunization with nanoparticles could obviously increase the production of lung T_EM_ cells, but the DCpep3-decorated nanoparticles could not enhance these effects further.

### DCs targeting nanoparticles significantly increased the generation of both CD4^+^ and CD8^+^ lung T_RM_ cells compared with the non-targeting protein

**Fig. 9:**
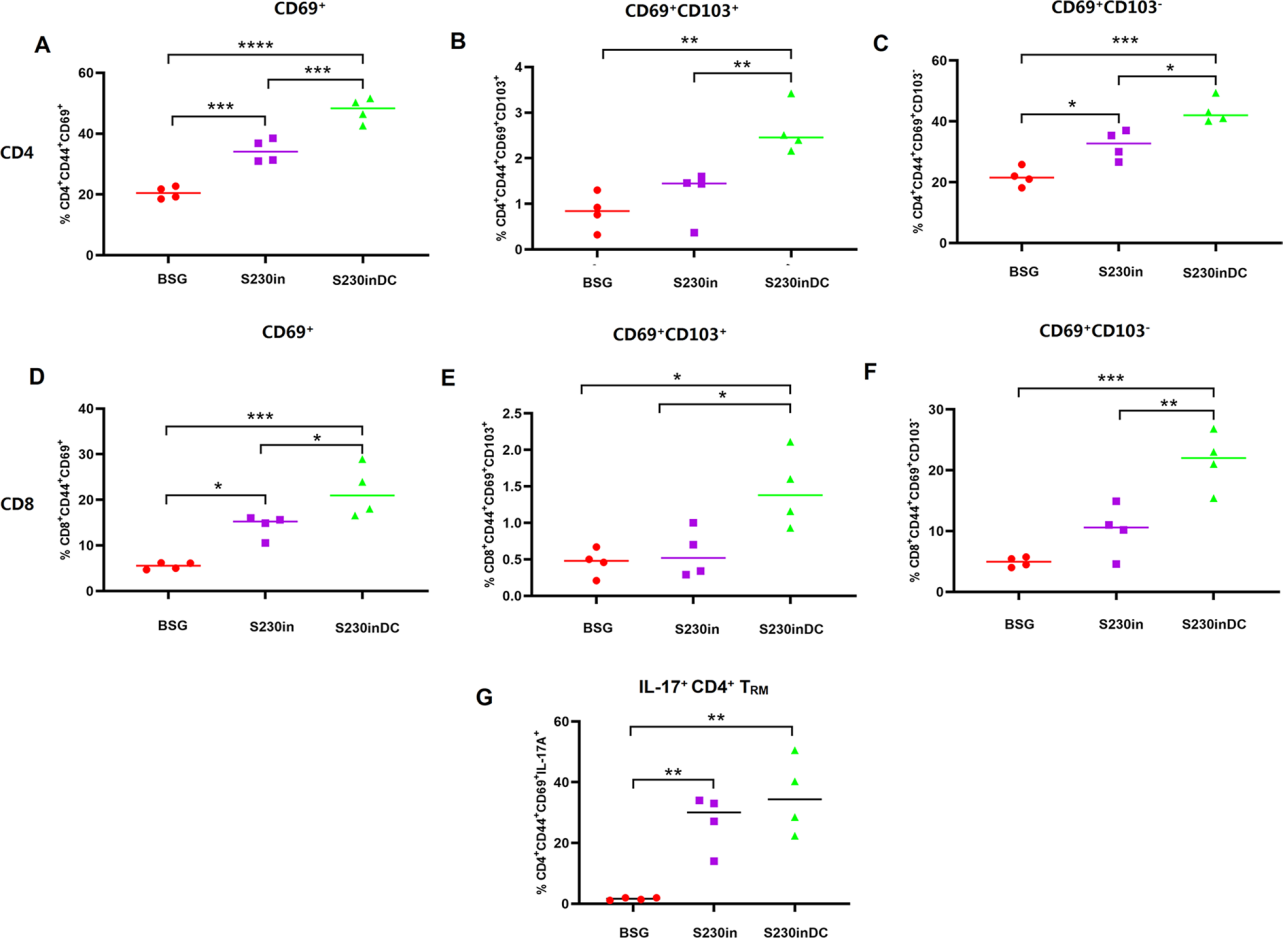
Generation of CD4^+^ and CD8^+^ lung-resident memory T (T_RM_) cells in lung tissues in Study 2. At 30 days post-3rd immunization in Study 2, single-cell suspensions from the lung samples were collected and subjected to FACS to determine the percentages of CD4^+^CD44^+^CD69^+^ (**A**), CD4^+^ CD44^+^CD69^+^CD103^+^ (**B**), CD4^+^ CD44^+^CD69^+^CD103^−^ (**C**), CD8^+^CD44^+^CD69^+^ (**D**), CD4^+^ CD44^+^CD69^+^CD103^+^ (**E**) and CD8^+^ CD44^+^CD69^+^CD103^−^ (**F**) T_RM_ cells. The intracellular production of IL-17 was also determined (**G**). (n = 4, *P < 0.05, **P < 0.01, ***P < 0.001)

The lung T_RM_ cells were finally determined by FACS assay, and the results showed that the CD4^+^CD44^+^CD69^+^ T cells were significantly increased by the non-targeting nanoparticle boost in the S230-primed mice (S230in) (Fig. 9A), consistent with previous results (Fig. 4A). Interestingly, intranasal boost immunization using DCs targeting nanoparticles (S230inDC) significantly increased the populations of CD4^+^CD44^+^CD69^+^ T cells further compared with those in the non-targeting group (*P* < 0.001) (Fig. 9A). In detail, both CD69^+^CD103^+^ (Fig. 9B) and CD69^+^CD103^−^ (Fig. 9C) CD4^+^ T cells were significantly increased in the S230inDC group compared with those in either the BSG control or S230in group. Similar results were also observed regarding lung CD8^+^ T_RM_ cells (Fig. 9D–F). On the other hand, FTY720 was administered to mice before intranasal boost immunization to prevent the recruitment of circulating lymphocytes into the lung. The results demonstrated that the presence of FTY720 severely affected the generation of lung CD4^+^ and CD8^+^ T_RM_ cells (Additional file 2: Fig. S2). No significant differences were observed among the BSG control, DC non-targeting and targeting groups with regard to CD4^+^ CD69^+^ (Additional file 2: Fig. S2A) and CD8^+^ CD69^+^ (Additional file 2: Fig. S2D) cells. An increasing trend of CD4^+^CD69^+^CD103^−^ (Additional file 2: Fig. S2C) and CD8^+^CD69^+^CD103^−^ (Additional file 2: Fig. S2F) T cells in the S230in and S230inDC groups were observed, where the percentages of CD4^+^CD69^+^CD103^−^ T cells in the S230inDC group were significantly increased compared with those in the BSG control (Additional file 2: Fig. S2C), indicating that there could be some “leaky” generation of lung T_RM_ cells even in the presence of FTY720. To further determine the potential immune function of lung T_RM_ cells, the intracellular production of IL-17A in CD4^+^ T_RM_ cells was also evaluated by flow cytometry. The results revealed that the percentages of CD4^+^CD44^+^CD69^+^IL-17A^+^ lung T_RM_ cells were significantly increased in both the S230in and S230inDC groups compared with those in the BSG control (Fig. 9G) upon stimulation with PMA, indicating that the generated lung T_RM_ cells could be functionally activated upon infection.

### Increased mucosal immune response and protection against virus challenge caused by DC targeting construction

**Fig. 10:**
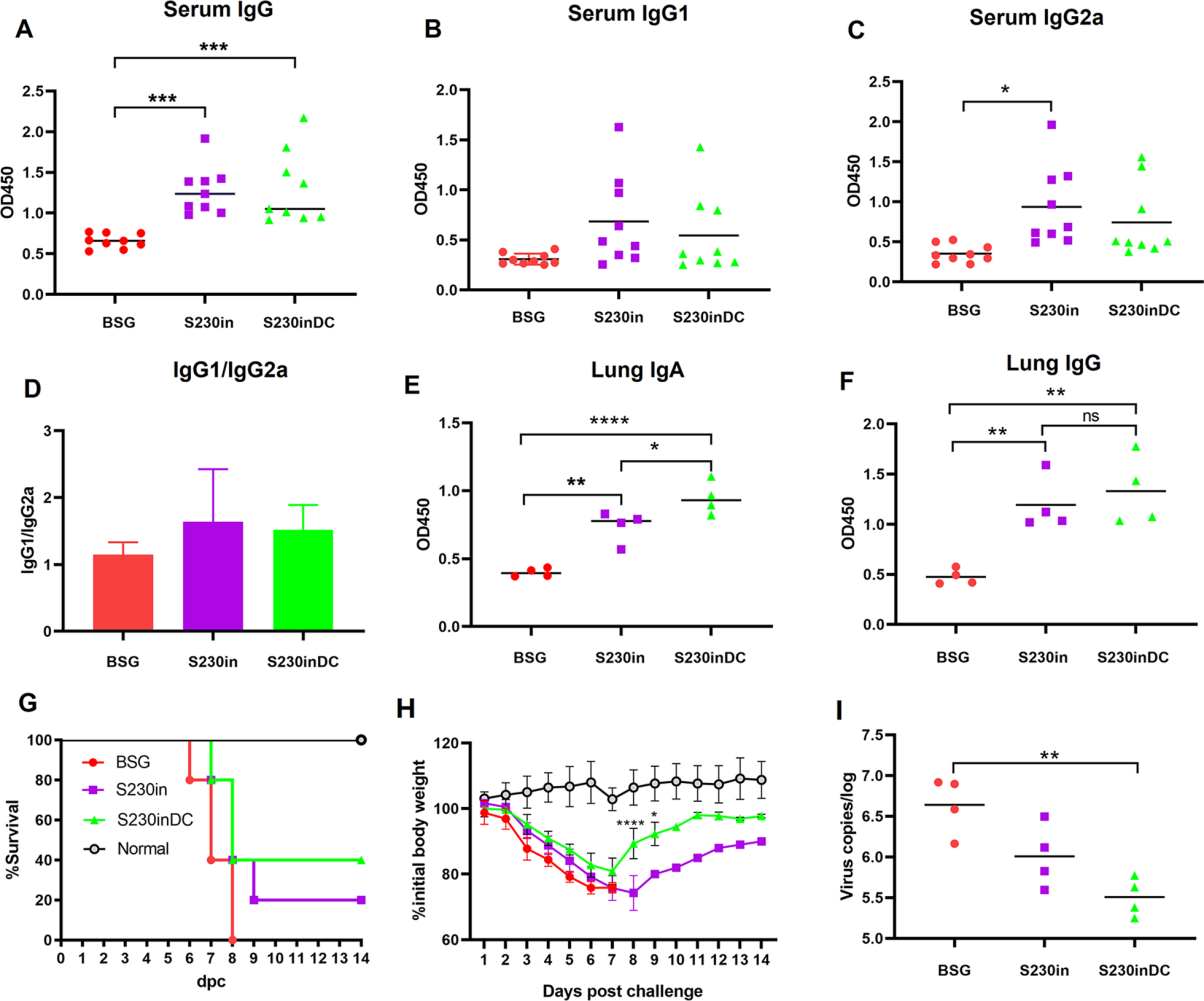
Measurement of M2e-specific antibodies and protection against influenza virus challenge in Study 2. ELISAs were performed to measure M2e-specific serum IgG (**A**), IgG1 (**B**), IgG2a (**C**) and the ratio of IgG1/IgG2a (**D**). The lung IgA (**E**) and IgG (**F**) antibodies were also determined. The mice were then challenged at a dose of 2 LD_50_ of H1N1 virus, and the survival rates (**G**) and body weight gains (**H**) were recorded for 14 days. On Day 5 post challenge, the lung samples were collected, and virus titers were determined by qRT‒PCR analysis (**I**). (n = 4, *P < 0.05, **P < 0.01, ***P < 0.001, ns, not significant)

To evaluate the humoral and mucosal immune response levels, serum (n = 9) and lung washing samples (n = 4) were collected on Day 10 post-3rd immunization (10 dp3i), and the M2e-specific antibody titers were determined by ELISA as described below. The results showed that the serum IgG levels in both S230in- and S230inDC-immunized mice were significantly increased compared with those in the BSG control (Fig. 10A). Then, the individual IgG1 (Fig. 10B) and IgG2a (Fig. 10C) antibodies and ratios of IgG1/IgG2a (Fig. 10D) were determined, which demonstrated that both IgG1 and IgG2a antibodies could be induced in these groups at very similar levels, indicating a relatively balanced Th1/Th2 immune response, with some Th2 bias observed. On the other hand, both S230in and S230inDC could stimulate elevated production of M2e-specific lung sIgA (Fig. 10E) and IgG (Fig. 10F) antibodies, in which the DC targeting construction could significantly increase the IgA antibody titers compared with those in non-targeting S230in (*P* < 0.05) (Fig. 10E). The results of antibody production demonstrated that intranasal boost immunization with DCpep3 nanoparticles had more effects on mucosal IgA antibodies than on humoral IgG antibodies compared with non-DC-targeting nanoparticles.

After challenge with the 2LD_50_ H1N1 strain, the mice in the BSG group began to die at 6 days post challenge (dpc), and all mice died at 8 dpc. On the other hand, although the mice in both the S230in and S230inDC groups also began to die at 7 dpc, the final survival ratios of these two groups were 20% and 40%, respectively, at the end of the study (Fig. 10G). The body weight gains during the 14-day challenge were also monitored, and the results showed that all the mice experienced dramatic body weight loss after challenge until 7 dpc, whereas the mice in the S230inDC group appeared to be less affected during the first 7 days and then recovered back more efficiently than the mice in the S230in group at 8 dpc (*P* < 0.001) and 9 dpc (*P* < 0.05) until the end of the study (Fig. 10H). To further evaluate the protective effects, the lung virus titers were determined at 5 dpc by qRT‒PCR assay (n = 4). The results demonstrated that compared with those in the BSG control, the virus titers in the S230inDC group appeared to decrease significantly (*P* < 0.01). Notably, a decreasing trend was also observed between S230in and S230inDC, although no significant difference could be calculated (Fig. 10I).

### Tail vein injection of sorted lung T_RM_ cells provides efficient protection against low-dose H1N1 challenge

**Fig. 11:**
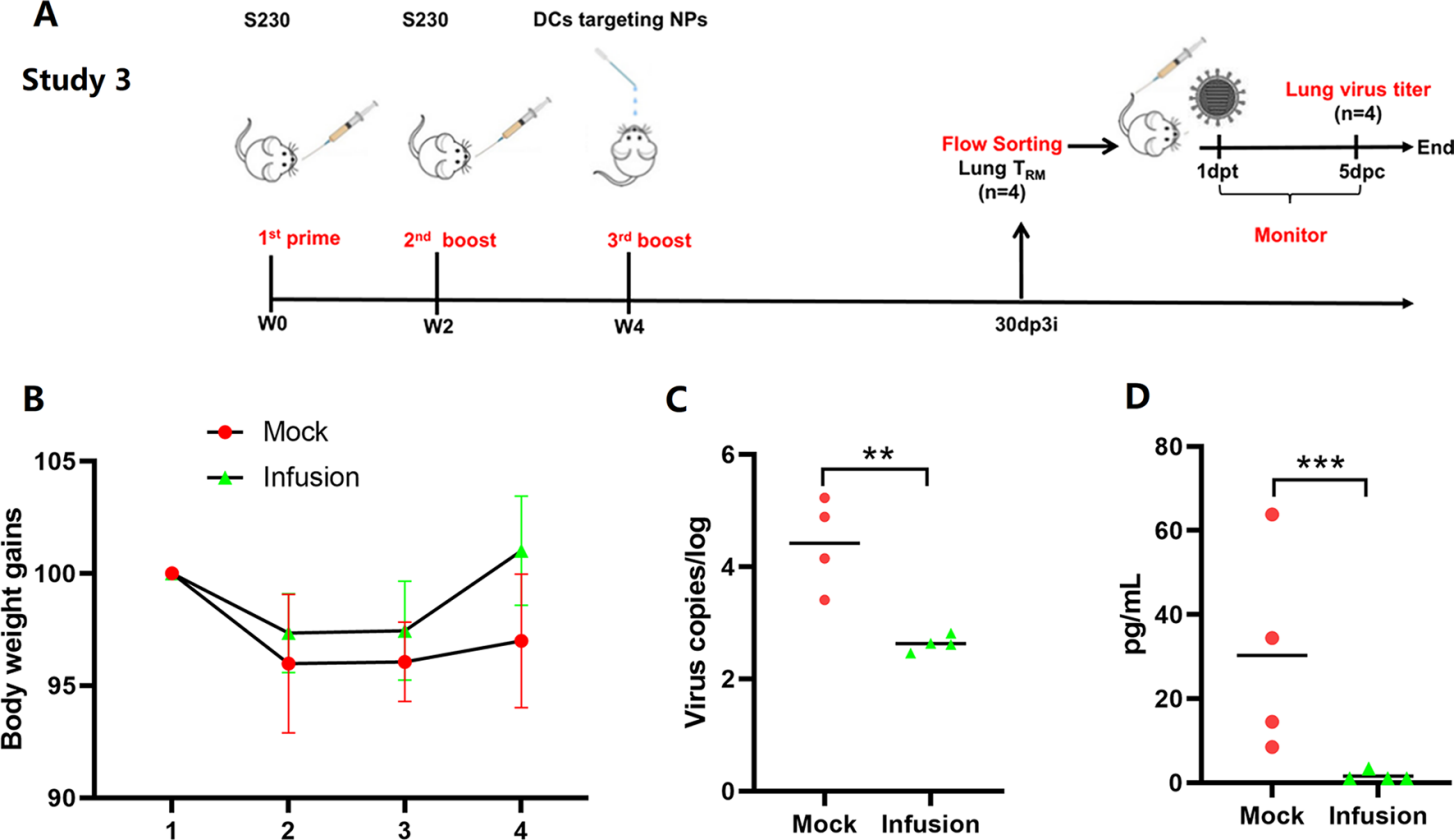
Protection against influenza virus challenge by transfer of T_RM_ cells in NOD/SCID mice. The 3rd animal study was designed in which lung T_RM_ cells were isolated from BALB/c mice at 30 days post the 3rd immunization mentioned in Study 2 by flow cytometry sorting and then transferred to NOD/SCID mice by tail vein injection (**A**). Mice without T_RM_ transfer were also included as mock controls. On the second day after transfer, low-dose H1N1 virus (0.1LD_50_) was used for challenge, and body weight gains (**B**) were recorded for 5 days. The virus titers were then determined by qRT‒PCR (**C**), and IL-6 production in lung washing samples (**D**) was measured by a LEGENDplex™ kit (Biolegend). (n = 4, **P < 0.01, ***P < 0.001)

To further determine whether the generated lung T_RM_ cells could play a role during the challenge, CD3^+^CD45^−^ lung T_RM_ cells were sorted by flow cytometry from BALB/c mice immunized in the S230in and S230inDC groups at 30 dp3i (n = 4) (Fig. 11A). Immunodeficient NOD/SCID mice, which have impaired T and B-cell lymphocyte development as well as defective natural killer cell function, were used as a receiver to evaluate the protective effects provided by T_RM_ cells. After tail vein injection treatment at a dose of 10^5^ cells/mouse, the mice were challenged with H1N1 on the second day at a dose of 0.1 LD_50_. The results showed that an obvious increase in body weight gain could be observed in the T_RM_-transferred mice compared with the mock control, although no significant difference could be calculated (Fig. 11B). The virus titers in lung samples were also determined by qRT‒PCR assay, and the results indicated that the transferred T_RM_ cells significantly decreased the virus titers compared with those in the mock control (*P* < 0.01) (Fig. 11C). In addition, the concentration of IL-6 in the lung washing collections was also measured by ELISA, and the results demonstrated that a dramatic decrease in the production of inflammatory IL-6 cytokines, compared with that in the mock group, could be observed in the presence of transferred T_RM_ cells (*P* < 0.001) (Fig. 11D). All the results mentioned above indicated that the transferred lung T_RM_ cells could provide efficient protection against challenge with a low dose of H1N1 virus, as expected.

## Discussion

It has been demonstrated that the enrichment of significant influenza-specific lung memory CD8^+^ T cells following influenza challenge in FTY720-treated or intranasally administered live attenuated influenza virus-vaccinated mice suggests in situ expansion of influenza-specific lung T_RM_ cells. Surprisingly, intranasal administration of inactivated influenza virus failed to elicit T-cell responses or provide protection against viral infection, demonstrating dual requirements for respiratory targeting and a live-attenuated strain to establish T_RM_ [22]. A similar result was also observed in which lung CD4^+^ and CD8^+^ T_RM_ cells significantly accumulated in the lungs of orally live attenuated *Yersinia pestis*-vaccinated mice and dramatically expanded, whereas short-term or long-term treatment of immunized mice with FTY720 did not affect lung T_RM_ cell formation and expansion or protection against pneumonic plague [42] since the *Yersinia pestis* strain could translocate into the lung by itself after oral immunization. All the results mentioned above indicated that the “alive” property of viruses or bacteria is critical to elicit in situ lung T_RM_ cells.

In our study, intranasal immunization with purified nanoparticles significantly increased the generation of lung T_RM_ cells in both the S180in and S230in groups, especially for S230 boost with either DC-nontargeting (Fig. 4) or -targeting nanoparticles (Fig. 9). Notably, our “non-alive” purified nanoparticles also did not induce the production of lung T_RM_ cells when FTY720 was administered before intranasal immunization (Additional files 1, 2: Fig. S1 and Fig. S2), which is consistent with the previous report showing that intranasal immunization with inactivated influenza virus did not elicit lung T_RM_ cells [22]. All the results mentioned above indicated that lung T_RM_ cells were not generated in situ in this study, most likely due to transfer from circulating lymphocyte organs to the lungs after intranasal immunization with purified nanoparticles. Interestingly, while oral administration of S230 stimulated an increased population of circulating T_EM_ cells in the spleen (Fig. 3), increased generation of lung T_RM_ cells was also observed after intranasal boost (Fig. 4), indicating that the elevated production of spleen T_EM_ cells could be responsible for the generation of lung T_RM_ cells.

In fact, it has been shown that circulating T_EM_ cells could be recruited to the lung, and some of them undergo phenotypic and functional conversion to T_RM_ cells [43], which is consistent with our hypothesis. A so-called “prime and pull” strategy has been developed to establish local T_RM_ cells at a site of potential viral exposure, in which conventional subcutaneous vaccination was first performed to elicit systemic T-cell responses (prime), followed by recruitment of activated T cells by means of topical chemokine application to the restrictive genital tract (pull), mediating excellent protection against genital herpes simplex virus 2 infection [44]. In detail, CXCR3 is upregulated on T cells upon activation and remains high through the effector and memory stages [45], and the application of chemokines CXCL9 and CXCL10 was sufficient to selectively recruit effector CD8^+^ T cells to the vagina through interaction with surface-displayed CXCR3, bypassing the requirement for CD4^+^ T-cell help. A similar “prime and pull” strategy was also reported in which the mice were immunized intramuscularly with Boostrix plus BcfA and boosted intranasally with the same formulation to generate IFN-gamma^+^ and IL-17^+^ CD4^+^ T_RM_ cells and significantly reduced *B. pertussis* colonization of the nose [46]. Other inflammatory agents [47] or the chemokine CCR5 [48] have also been found to sufficiently generate CD8^+^ T_RM_ cells in the skin or gut.

A similar “prime and pull” strategy could therefore be at least one of the possible explanations for the increased production of lung T_RM_ cells by dual DC-targeting vaccine design in this study (Additional file 3: Fig. S3). The oral administration of scFv-CD11c-decorated *Salmonella* could efficiently increase the generation of circulating CD4^+^ and CD8^+^ T_EM_ cells (Fig. 3), and the hypothesis was that intranasal boost immunization could release some specific cytokines or chemokines that would recruit circulating T_EM_ cells to the lung and then transform them to T_RM_ cells. In fact, intranasal immunization using DC non-targeting and targeting nanoparticles significantly increased the production of IL-17 in lung CD4^+^ T cells compared with that in the BSG control, especially DCs targeting nanoparticles (Fig. 6C). Notably, Th17 and IL-17 have already been reported to be involved in the recruitment of neutrophils and other immune cells to lung or infection sites mediated by chemokines induced by IL-17 [49, 50]. In addition, IL-17 has also been reported to be a critical factor during the formation and maintenance of lung T_RM_ cells [42, 51, 52]. Therefore, it would be reasonable to deduce that the increased production of IL-17 in the S230inDC group could be responsible for the elevated generation of lung T_RM_ cells compared with that in both the BSG control and DC non-targeting groups (Fig. 9). Notably, a number of chemokines, such as CXCL13 and CXCL15, were demonstrated to be upregulated in lung lymphocytes isolated from mice in the S230inDC group at 10 dp3i compared with those in the S230in group by RNAseq as well as qRT‒PCR assays (Fig. 7), which could play a critical role during the formation of lung T_RM_ cells together with IL-17. Interestingly, it was reported that the “prime and pull” strategy based on chemokines CXCL9 and CXCL10 only established CD8^+^ T_RM_ cells but not CD4^+^ T_RM_ cells [44]. In contrast, our “oral immunization prime followed by intranasal boost pull” strategy resulted in significantly increased production of both CD4^+^ and CD8^+^ lung T_RM_ cells (Figs. 4, 9), possibly because a number of cytokines or chemokines other than CXCL9 and CXCL10, such as type I interferons [53], TGF-beta [15], IL-7 [54], IL-15 [55] and IL-21 [56], which have been reported to be involved in the recruitment and transfer of T_EM_ cells or maintenance of T_RM_ cells, were also induced by our intranasal boost. However, the crosstalk between the spleen-lung axis has not been clearly elucidated yet and still need to be addressed in future. Another possible piece of evidence indicating that the lung T_RM_ cells were mostly converted from circulating T_EM_ cells instead of locally generated from lung T_EM_ cells was that intranasal immunization yielded very similar levels of lung T_EM_ cells in mice orally administered S180 and S230 (Fig. 3D, E), whereas significantly increased production of lung T_RM_ cells in S230-primed mice was observed compared with that in the S180 group (Fig. 4). Notably, the underlying molecular mechanisms of how scFv-CD11c-decorated *Salmonella* could result in an increased population of spleen T_EM_ cells still need to be uncovered in the future.

However, it should be noted that a relatively small portion of lung T_RM_ cells were transformed locally from other immune cells, such as Th17 cells, in this study (Additional file 3: Fig. S3). Pretreatment with FTY720 dramatically decreased the generation of lung T_RM_ cells in mice primed with either S180 or S230 followed by intranasal boost using DC nontargeting nanoparticles (Additional file 1: Fig. S1). On the other hand, there was also increased production of lung T_RM_ cells in mice primed with S230 followed by intranasal boost using DCs targeting nanoparticles, especially CD4^+^CD69^+^CD103^−^ T_RM_ cells, compared with that in the BSG control (Additional file 2: Fig. 2C), indicating that some locally produced T_RM_ cells could be present. In fact, a previous report noted that a significant fraction of lung CD4^+^IL17^+^ T_RM_ cells were derived from IL-17A-producing effector (Th17) cells following immunization with heat-killed *Klebsiella pneumonia* [54]. Therefore, a possible explanation for the locally generated lung T_RM_ cells in S230inDC-immunized mice could be that DC-targeting nanoparticles could elicit the production of Th17/IL-17 more efficiently than nontargeting DCs (Fig. 6C).

A DC targeting strategy has previously been used as an efficient approach to enhance the generation of lung T_RM_ cells [15]. “Antibody-targeted vaccination” involves the inoculation of antigen coupled to monoclonal antibodies (mAbs) against DC receptors. Two major DC populations within the murine lung have been identified as CD11b^+^ and CD103^+^ DCs, respectively, with different expression levels of the surface markers DEC205 and Clec12A. The intranasal delivery of anti-Clec12A antibody could label both DC subsets within the lung, whereas the intranasal delivery of anti-DEC205 antibody labeled only the CD103^+^ DC subset. In addition, both CD11b^+^ and CD103^+^ DCs could cross-present model antigen-OVA protein, especially CD103^+^ DCs, which efficiently promoted CD103 upregulation upon CD8 T-cell activation in a TGFβ-dependent manner. Notably, TGFβ has been considered to be a cytokine that promotes CD103 expression and converts cytotoxic T lymphocytes into lung T_RM_ cells. By conjunction with anti-DEC205 or anti-Clec12A antibodies, the OVA protein could provide efficient protection against influenza challenge, especially in the anti-DEC205 antibody fusion construct, indicating that CD103^+^ DCs could be a more important subtype of lung DCs than CD11b^+^ DCs. Contrary to these findings, we have identified that intranasal immunization using DCs targeting peptide 3-decorated nanoparticles in our study would prefer to be captured and stimulate the maturation of CD11b^+^ DCs, instead of the CD103^+^ subtype (Fig. 5D). Unexpectedly, there was even an obviously decreased percentage of CD103^+^ DCs after intranasal immunization with ferritin nanoparticles. The involvement of CD11b^+^ DCs in our study was probably caused by the different targeting mechanisms between DCpep3 and the anti-DEC205 antibody.

Notably, the production of IL-17 not only appeared to be involved in the generation of lung T_RM_ cells but also played a critical role in T_RM_ cell-mediated protection against infection, especially CD4^+^ T_RM_ cells. For example, CD4^+^IL-17^+^ T_RM_ cells have been demonstrated to be critical for the clearance of *B. pertussis* [57, 58], pneumonic *Yersinia pestis* infection [42], *Mycobacterium tuberculosis* [59] and *Klebsiella pneumonia* [52]. Similar results were also observed in CD8^+^ T_RM_ cells, which showed that the CD8^+^ T_RM_ cells in patients with erosive oral lichen planus displayed enhanced cytokine production, including IFN-gamma, TNF-a and IL-17 [60]. Consistent with previous reports, the lung CD4^+^ T_RM_ cells induced by intranasal boost immunization with both DC non-targeting and targeting nanoparticles in this study also significantly increased the production of IL-17 (Fig. 9G). IL-17A acts on non-immune cells in infected tissues to strengthen innate immunity by inducing the expression of antimicrobial proteins, cytokines, and chemokines and recruiting neutrophils and macrophages into the airways to stimulate successful host defense against pathogens [61]. Therefore, the increased generation of IL-17 by lung CD4^+^ T_RM_ cells was probably one of the possible factors responsible for enhanced protection against influenza virus challenge.

During the “prime and pull” strategy, the pull time was another interesting issue to consider for efficient production of T_RM_ cells. A previous study indicated that the chemokine pull was most effective at recruiting recently activated effector CD8^+^ T cells that are circulating at high frequency [44], establishing a specific time frame within which the chemokine pull should be administered after priming. After comparing the chemokine pull at the effector (d5), contraction (d15) and memory (d30) phases of the T-cell response [62], it was found that the chemokine pull was most effective at recruiting antigen-specific CD8^+^ T cells during the effector (d5) phase [44]. Since our intranasal boost was performed on Day 14 after the 2nd immunization, close to the contraction phase of the T-cell response, but not the best effector phase as mentioned above, it will be meaningful to perform the intranasal boost on Day 5 after the last oral administration to determine whether the boost could enhance the generation of lung T_RM_ cells in the future.

On the other hand, it is worth mentioning that the presence of general lung T_RM_ cells instead of antigen-specific lung T_RM_ cells was determined in this study, therefore, it would be more convincing to perform an MHC tetramer assay to illustrate the role of M2e-specific T_RM_ cells in the future. Another potential weakness of this study is that protection against different heterologous influenza virus challenges was not included to determine whether the M2e-based conserved nanoparticles could be a universal vaccine candidate, which could be confirmed in the future through the adoptive transfer of M2e-specific T_RM_ cells in NOD/SCID mice and challenge with heterologous virus. In addition, the inclusion of some other better protective antigens such as HA and NA could possibly improve the vaccine efficiency further in future study.

## Conclusion

In conclusion, we designed a novel immunization strategy to enhance the production of lung T_RM_ cells based on a combination of two DC targeting designs mediated “prime and pull” strategy, including scFv-CD11c-decorated *Salmonella* and DC-targeting peptide-decorated ferritin nanoparticles. The surface-displayed scFv-CD11c on *Salmonella* could efficiently increase the amount of circulating T_EM_ cells after oral administration, whereas intranasal boost immunization using purified DCs targeting nanoparticles could dramatically recruit the circulating T_EM_ to the lung, yielding higher percentages of T_RM_ cells protective against influenza virus.

## Experimental section

### Bacterial strains and virus used

*Escherichia coli* (*E. coli*) χ6212 (*asd* mutation), Top10, BL21, *Salmonella* χ11802 [39] and the original plasmid pYA3681 for molecular cloning used in this study were generously provided by Roy Curtiss III (University of Florida, USA), as shown in Table 1. pET28a (Invitrogen) was used for protein expression and purification. Luria–Bertani medium (LB) was used for bacterial culture at 37 °C, and when necessary, 50 μg/mL diaminopimelic acid (DAP) (Sigma), 0.1% arabinose (Sigma), 0.2% mannose (Sigma) and 50 μg/mL kanamycin were provided. For the influenza virus challenge study, the H1N1 virus (A/Puerto Rico/8/1934) [63] was used.

**Table 1:** Bacterial strains, plasmids and virus used

Materials	Description	Source
Bacteria strains		
χ11802	Regulated delayed lysis *Salmonella*	Roy Curtiss III University of Florida
χ6212	*E.coli* host strain with asd mutation	Roy Curtiss III University of Florida
Top10	*E.coli* host strain for regular cloning	Kangti, China
BL21	*E.coli* host strain for protein expression and purification	Kangti, China
Plasmids		
pYA3681	Prokaryotic expression vector in *Salmonella*, arabinose dependent	Roy Curtiss III University of Florida
pYL180	pYA3681 based prokaryotic vector, express 3M2e-ferritin fusion protein	Wang et al. [38]
pYL230	pYL180 based prokaryotic vector, express 3M2e-ferritin fusion protein, as well as pgsA’-scFv-CD11c sequence	This study
pYL139	PgsA’ anchored scFv-CD11c on surface of *E.coli* or *Salmonella*	This study
pET28a	Prokaryotic expression vector, kanamycin resistant	Lab stock
pYL272	pET28a based vector expressing 3M2e-ferritin, His tag	This study
pYL273	pET28a based vector expressing DCpep3-3M2e-ferritin, His tag	This study
pYL285	pEASY-blunt based standard plasmid harboring M1 gene of H1N1	Lab stock

The virus was prepared in the allantoic cavity of 9- to 11-day-old specific pathogen-free eggs from Harbin Veterinary Research Institute (Harbin, China), and the virus titer was determined in female BALB/c mice and expressed as the 50% lethal dose (LD_50_) by the Reed and Muench method [64].

### Molecular manipulation

In our previous study, we constructed a *Salmonella*-based prokaryotic expression vector, pYA180 [38], which could synthesize three copies of M2e (MSLLTEVETPIRNEWGCRCNGSSD) (3M2e) of H1N1 virus fused with ferritin protein, which could self-assemble into a 24-mer nanocage. Plasmid pYL139, which contained the pgsA’ anchoring sequence [32], and scFv-CD11c [31] were synthesized by GenScript (Suzhou, China). Primers 139-F-SacII/139-R-XbaI were used to amplify the pgsA’-scFvCD11c fragment, as well as the transcription terminal sequences and additional Shine‒Dalgarno (SD) sequences ahead of the start codon ATG of the pgsA’ cassette, using plasmid pYL139 as a template. The fragment was then digested by SacII/XbaI and ligated with plasmid pYL180 digested using the same enzymes, yielding pYL230, which could express 3M2e-ferritin and scFv-CD11c at the same time (Fig. 1A).

The plasmid pET28a was used to construct the prokaryotic expression vector for intranasal immunization. In detail, primers 272-F-NdeI/ferritin-R-BamHI and 273-F-NdeI/ferritin-R-BamHI were used to amplify the 3M2e-ferritin fragment using pYL180 as a template. After double digestion using NdeI and BamHI, the fragments were ligated with pET28a digested with the same enzymes, yielding pYL272 and pYL273, respectively. Notably, pYL273 was designed to harbor a short DCpep3 sequence ahead of the 3M2e-ferritin fragment (Fig. 5A).

### Determination of protein synthesis

The constructed individual plasmid was transformed into *Salmonella* strain χ11802 to evaluate its expression. The strains were cultured in LB medium supplemented with 0.1% arabinose and 0.2% mannose overnight at 37 °C with shaking. Then, the bacteria were inoculated into fresh medium at a dilution of 1:100 and cultured further until the OD_600_ reached 0.6, when 1 mM isopropyl β-d-thiogalactoside (IPTG) (Beyotime Biotechnology, China) was added and incubated for an additional 16 h. Then, the bacteria were collected by centrifugation at 12,000 rpm for 10 min, and the purification of 24-mer ferritin nanocage was performed as described previously [38]. The purified nanoparticles were then analyzed by both reducing and non-reducing western blotting, in which the mouse monoclonal anti-influenza A virus M2 protein IgG antibody (14C2) (1:1000, Abcam, UK) was used as the primary antibody, followed by HRP-labeled secondary antibodies (Proteintech, China). The nanoparticles were also characterized using transmission electron microscopy (TEM) and immune electron microscopy (Harbin Veterinary Research Institute, Harbin, China) to confirm the presence of 3M2e on the surface of the ferritin nanoparticles as described previously [38].

To determine the surface-displayed scFv-CD11c (His tag) on *Salmonella*, the cell wall components were collected using a commercial bacterial outer membrane protein extraction kit (BBproExtra, Bestbio, China) according to the instructions. The extracted proteins were then subjected to western blot using the mouse monoclonal anti-His tag antibody (Proteintech, China) and HRP-labeled rabbit anti-mouse IgG antibody (Proteintech, China) as primary and secondary antibodies, respectively. The surface-located scFv-CD11c was also determined by the indirect immunofluorescence assay (IIF) as described previously [65] with minor modifications. In detail, *Salmonella* χ11802 harboring either pYL180 or pYL230 was induced by 1 mM IPTG for 4 h at 37 °C with shaking, and then 1 mL of bacterial culture was centrifuged at 16,000×*g* for 10 min to collect the pellet. After washing twice with PBS, the bacteria were resuspended and incubated with 1 mL PBS with mouse monoclonal anti-His tag antibody (Proteintech, China) at a dilution of 1:400 overnight at 4 °C. Then, the bacteria were washed three times with PBS and incubated with FITC-conjugated goat anti-mouse IgG antibody (Beyotime, China) at a dilution of 1:100 in PBS for an additional 1 h. After an additional three washes with PBS, the bacterial pellets were resuspended in 100 µL PBS and analyzed by a Leica DM18 microscope (Germany) with a 100× oil immersion optic.

### Determination of BMDC cellular uptake by scFv-CD11c-anchored *Salmonella*

BMDCs were prepared as described previously [66] with minor modifications using GM-CSF (20 ng/mL) and IL-4 (20 ng/mL) and then incubated with S246 and S256 to evaluate whether the presence of scFv-CD11c could enhance the cellular uptake efficiency. In detail, BMDCs were inoculated into a 24-well plate with 2 × 10^5^ cells/well on Day 8 and cultured overnight. The *Salmonella* strains S246 and S256 were cultured until OD600 reached approximately 0.6, and then 1 mM IPTG was added and incubated for an additional 4 h. Then, the bacteria were collected by centrifugation and adjusted to 2 × 10^7^ CFU/mL, in which 100 µL bacterial suspensions were added to each well and incubated with BMDCs for 2 h. After that, the cells were fixed with 4% formaldehyde for 20 min at room temperature, followed by DiI staining for the cell membrane and DAPI staining for the nucleus, according to the instructions. The cells were then analyzed by a Leica SP5 confocal microscope (Germany).

The cellular uptake efficiency was also determined by flow cytometry as described above with minor modifications. The BMDCs were incubated at 2 × 10^6^ cells/well in a 6-well plate and then stimulated with S246 and S256 individually with a multiplicity of infection (MOI) of 100. After 2 h of co-incubation, the cells were collected by centrifugation and subjected to flow cytometry analysis by incubation with APC-conjugated anti-mouse CD11c antibody (BD Bioscience) for 30 min at 4 °C. The EGFP-positive cells in the cluster of CD11c^+^ BMDCs as well as the mean fluorescence intensity (MFI) were calculated.

### Determination of BMDCs cellular uptake by DCpep3-decorated nanoparticles

The purified nanoparticles were stained using Flamma 496 NHS (Bioacts, Korea) according to the manufacturer’s instructions. The BMDCs were prepared as above and inoculated into a 24-well plate with 2 × 10^5^ cells/well. The stained protein nanoparticles were then added at the same time at a final concentration of 40 μg/mL and incubated for 16 h. After that, the cells were stained with a mouse anti-LAMP1 antibody (Proteintech, China), followed by Cy3-labeled goat anti-mouse IgG antibody (Beyotime, China) as a secondary antibody and analyzed by confocal microscopy.

### BMDC maturation caused by scFv-CD11c *Salmonella*

The *Salmonella* strains S246 and S256 were incubated with BMDCs as described above using an MOI of 100 for 2 h, and BSG and LPS (0.5 μg/mL) were also included as negative and positive controls, respectively. After washing with PBS 3 times, the cells were incubated with complete culture medium containing 100 μg/mL gentamicin for an additional 12 h. After that, the cells were collected and subjected to flow cytometry analysis using antibodies against APC-mouse CD11c, FITC-mouse CD40, PE-mouse CD86 and percp-cy5.5-mouse MHC-II (BD Bioscience).

### Mouse study design

All animal procedures were approved by the Institutional Animal Care and Use Committee at Jilin Agriculture University (2023 02 15 001). In general, three mouse studies were designed for different purposes:

Study 1: The first study was intended to address the effects of scFv-CD11c on the production of T_EM_ cells. A total of 64 6- to 8-week-old pathogen-free BALB/c female mice were purchased from Beijing HFK Bioscience Co., Ltd., China and randomly divided into 5 groups with 16 mice in the BSG, S180 and S230 groups and 8 mice in the S180 and S230 groups. The mice were orally administered S180 or S230 at a dose of 1.0 × 10^9^ CFU/0.1 mL BSG on Days 0, 14 and 28. A third dose intranasal immunization with 10 µg of 3M2e-ferritin nanoparticles purified from 1.0 × 10^9^ CFU *Salmonella* in a volume of 20 µL was also performed on Day 28 in mice previously treated with two oral administrations of S180 and S230 at Days 0 and 14, designated S180in and S230in, respectively. Mice treated with BSG buffer were also included as a negative control. On Day 1 post-2nd immunization (dp2i), 4 mice from the BSG, S180 and S230 groups were sacrificed, and the PP, MLN and spleen samples were collected and analyzed by flow cytometry to determine the maturation of DCs by evaluating MHC-II, CD40 and CD86. At 10 dp2i, spleen samples were collected and subjected to FACS assay to determine the percentages of T_EM_ cells. At 10 dp3i, spleen and lung samples were also collected from all groups and analyzed by flow cytometry to evaluate the production of T_EM_ cells. Then, lung samples from all groups were collected at 30 dp3i to determine the percentages of lung T_RM_ cells.

Study 2: The 2nd mouse study was designed to compare the difference between mice primed with orally administered S230, followed by intranasal boost immunization using nanoparticles purified from either DCs targeting (pYL273) or non-targeting (pYL272) construction. A total of 72 6- to 8-week-old specific pathogen-free female BALB/c mice were randomly divided into 3 groups (n = 24/group), including the BSG control group, *Salmonella* χ11802 (pYL230) oral immunization followed by intranasal boost with 10 µg of non-DC-targeting nanoparticles group (named S230in) and DC-targeting nanoparticles group (named S230inDC). At 1 dp3i, 4 mice from each group were sacrificed by CO_2_ euthanasia, and single-cell lymphocytes were prepared from lung samples as described previously [67]. Then, the activation of lung CD103^+^ and CD11b^+^ DCs was assayed by FACS targeting the surface markers MHC-II and CD80. At 10 dp3i, intracellular cytokine production (IL-4, IFN-gamma, IL-17, IL-2 and TNF-alpha) in lung and spleen lymphocytes was determined by flow cytometry or ELISpot assay as described below. In addition, the percentages of T_EM_ cells in both the spleen and lung were also evaluated by flow cytometry. At the same time, lung samples from each group were also collected and subjected to RNAseq analysis by Novagene (China). qRT‒PCR assays were then performed to confirm the RNAseq results. At 30 dp3i, the lung single-cell lymphocytes were collected and subjected to FACS assay to determine the production of T_RM_ cells and the intracellular production of IL-17 in T_RM_ cells upon stimulation with PMA. Serum IgG and lung IgG/IgA antibody titers were also measured by ELISA. At 35 dp3i, the remaining mice were challenged at the dose of 2 LD_50_ of A/Puerto Rico/8/1934 (H1N1) virus, and the survival curve as well as body weight gains were recorded for 14 days. At 5 days post challenge (dpc), 4 mice from each group were sacrificed, and lung samples were collected to determine the lung virus titers by qRT‒PCR.

Study 3: The 3rd mouse study was designed to evaluate the protective effects of sorted total T_RM_ cells from mice in the S230inDC group. A total of 8 NOD/SCID mice were divided into two groups with 4 mice per group. CD45^−^CD3^+^ lung T_RM_ cells were isolated by flow sorting and given back to 4 NOD/SCID mice by tail vein injection at a dose of 10^5^ live cells/mouse as a T_RM_ group. The remaining mice were treated the same way using PBS as a negative control. One day later, H1N1 virus was used for challenge at a dose of 0.1 LD_50_, and the clinical signs and body weight gains were monitored for 5 days. Then, the mice were sacrificed, lung samples were collected, and the virus titers were determined by qRT‒PCR assay. Production of inflammatory cytokine IL-6 was also determined by a LEGENDplex™ kit (BioLegend).

### FTY720 treatment

FTY720 dissolved in water (1.85 mg/L) was supplied ad libitum to the mice. Mice received an estimated dosage of 1.25 mg/kg/Day 7 days before intranasal boost immunization and lasted for 30 days until tissues were harvested. Water was changed at least once a week [68].

### RNAseq analysis

Lung samples were collected at 10 days post-3rd immunization in Study 2, and total RNA was isolated using TRIzol (Invitrogen) according to the manufacturer’s protocol. RNAseq was performed by Novagene (China), and heatmap clustering and KEGG enrichment analysis were then conducted. Differential expression analysis of two conditions/groups (two biological replicates per condition) was performed using the DESeq2 R package (1.20.0). The resulting P values were adjusted using Benjamini and Hochberg’s approach for controlling false discovery rate. Genes with an adjusted P value <  = 0.05 found by DESeq2 were assigned as differentially expressed. KEGG analysis was performed using the clusterProfiler R package to test the statistical enrichment of differentially expressed genes. qRT‒PCR assays with primers CXCL13-F/R and CXCL15-F/R (Table 2) were then used to confirm the RNAseq results, and GAPDH was also included as an internal standard.

**Table 2:** Primers used in this study

Primers	Sequences
139-F-SacII	gcgCCGCGG*aggaaacagacc*ATGAAGAAAGAATTAAGTTTCC
139-R-XbaI	agaTCTAGACTGTCAGACCAAGTTTACTC
272-F-NdeI	agcCATATGATGAGCCTGCTGACTGAAGTTG
273-F-NdeI	agcCATATG*TCATTGAGTCTATTAACAATGCCCGGAAATGCGAGC*ATGAGCCTGCTGACTGAAGTTG
ferritin-R-BamHI	cacGGATCCTTAGCTTTTGCGGCTTTTCG
H1N1-M1F	AGGCCAAATGCCACTTCAG
H1N1-M1R	AGGAAGCTCAAGAGGGAGATAACA
GAPDH-F	CCCTTCATTGACCTCAACTACA
GAPDH-R	ATGACAAGCTTCCCGTTCTC
CXCL13-F	GGCCACGGTATTCTGGAAGC
CXCL13-R	ACCGACAACAGTTGAAATCACTC
CXCL15-F	TCGAGACCATTTACTGCAACAG
CXCL15-R	CATTGCCGGTGGAAATTCCTT

Underlined: enzyme site; italic lowercase: SD sequence; italic capital letter: DCpep3 sequence

Wang et al. [38]

### Flow cytometry analysis

Single-cell suspensions were prepared from the spleen and lung tissues as described before [67] with minor modifications. In detail, spleens were collected and gently crushed in complete RPMI 1640 containing 10% heat-inactivated FCS (Sigma), 50 mg/mL streptomycin and 50 U/mL penicillin (HyClone). For isolation of lymphocytes from lung sections, lung tissues were digested with 250–300 U/mL collagenase type IV (Sigma) in complete RPMI 1640 for 30 min at 37 °C and then filtered through a 70 μm filter. The cells from the spleen and lung were then resuspended in red blood cell lysis buffer (Solarbio, Beijing, China) for 2 min at room temperature. After washing twice using RPMI medium, the cells were diluted to the desired concentration. FACS was then performed using BD Fortessa with specific antibodies from Biolegend, except for a few from BD Biosciences, as indicated. The detailed gating strategies were included as shown (Additional file 4: Fig S4, Additional file 5: Fig. S5. Additional file 6: Fig. S6, Additional file 7: Fig. S7, Additional file 8: Fig. S8).

During the study, a Zombie NIR™ Fixable Viability Kit (Biolegend) was used to characterize live cells, and TruStain FcX™ (Biolegend) was employed to block Fc receptors if necessary. To determine the production of T_EM_, T_CM_ and T_Naive_ cells, antibodies including PerCP/Cyanine5.5 anti-mouse CD3ε (BD Biosciences, USA), FITC-anti-CD4 antibody, Alexa Fluor® 700-anti-CD8α antibody, PE/Cyanine7-anti-mouse/human CD44, and APC-anti-mouse CD62L were selected. To evaluate the maturation of DCs, antibodies including Alexa Fluor® 700-anti-mouse F4/80, APC-anti-mouse CD11c, Cyanine5.5-anti-mouse I-A/I-E, PE-anti-mouse CD86, FITC-anti mouse CD40, PE/Cyanine7-anti-mouse CD11b and PE-anti-mouse CD103 were used. To determine the generation of T_RM_ cells in lung sections, mice were intravenously injected with 3 μg of APC/Cy7 anti-mouse CD45 (BioLegend) diluted in 300 μL of saline 5 min before euthanasia [69]. After that, single-cell suspensions were prepared as mentioned above, and antibodies including PerCP/Cyanine5.5 anti-mouse CD3ε (BD Biosciences, USA), FITC-anti-CD4 antibody, Alexa Fluor® 700-anti-CD8α antibody, PE/Cyanine7-anti-mouse/human CD44, PE-anti-mouse CD69 and APC-anti-mouse CD103 were used.

To quantify the production of intracellular cytokines from antigen-specific T cells, single cell suspensions were prepared from spleen or lung as mentioned above and treated with 5 μg/mL synthesized H1N1 M2 peptide (GenScript, China) and 2 μg/mL anti-CD28 (BioLegend) at 37 °C with CO_2_ for 1 h. Cells were then incubated with Protein Transport Inhibitor (BD Biosciences, USA) for another 5 h to block the secretion of cytokines. DMSO and PMA/ionomycin were used as negative and positive controls, respectively, when necessary. After a total of 6 h, the cells were incubated with a fixation/permeabilization kit (BD Biosciences, USA), and antibodies including PE-anti-IL-4 antibody, APC-anti-IFN-γ antibody, and PE/Cyanine7-anti-mouse TNF-α, BV421-anti-mouse TNF-α, PE/Cyanine7-anti-mouse IL-17a and PE-CF594-anti-mouse IL-2 were selected. Data compensation and analysis were performed using FlowJo Version 7.6.1.

### ELISpot analysis

IFN-gamma-producing lymphocytes were evaluated according to the instructions (Mabtech, Sweden). In detail, the spleens were collected at 10 dp3i from each group (n = 4), and single lymphocytes were prepared as mentioned above. Then, the cells were diluted and added to each well coated with IFN-gamma capture antibodies at a density of 2.0 × 10^5^ cells/well. After incubation with 5 mg/mL M2e peptides for 30 h, the lymphocytes were removed, and IFN-gamma secretion was detected using an ImmunoSpot Analyzer by CTL (Cellular Technology Ltd.) to calculate the spot forming units (SFU).

### ELISA to determine M2e-specific antibody titers

ELISA was performed as described previously [65] to evaluate the M2e-specific antibody response. In detail, 96-well ELISA plates (NEST, China) were coated with synthesized M2e peptide (MSLLTEVETPIRNEWGCRCNGSSD) from the H1N1 influenza virus (GenScript, China) at a concentration of 500 ng/well in coating buffer (pH 9.6) at 4 °C overnight. After washing three times with PBS buffer containing 0.05% tween-20 (PBST) and blocking with PBST-BSA overnight at 37 °C, the serum (1:100) and lung washing samples (1:20) were added to each well with triplicate repeats. Then, biotin-labeled anti-mouse IgG, IgG1, IgG2a and IgA antibodies (Southern Biotechnology, Birmingham, AL) diluted 1:10,000 and streptavidin-HRP (Southern Biotechnology, Birmingham, AL) diluted 1:3000 were added, followed by tetramethylbenzidine (TMB) substrate to develop color and recorded at 450 nm.

### qRT‒PCR assay to determine the lung virus titers

At 5 dpc, the lungs were removed and subjected to determination of viral load by qRT‒PCR using the standard plasmid pYL285 and primers H1N1-M1F/M1R. Total RNA was extracted using an RNA isolation kit (Beyotime Biotechnology, China), and 1 μg of RNA was reverse transcribed to cDNA in a total reverse-transcription system of 20 μL with a BeyoRT™ First Strand cDNA Synthesis Kit (Beyotime Biotechnology, China). Then, 2 μL of the final obtained cDNA was aspirated for quantitative PCR by an ABI7500 Real-Time PCR system. The mRNA levels of GAPDH were also determined using the primers GAPDH-F/GAPDH-R as an internal control.

### Statistical analysis

All data are displayed as the mean ± SD. Prism 8.3 (GraphPad) was used to perform one-way ANOVA and Tukey’s posttest for statistical analysis (*P* < 0.05 was considered to be a significant difference).

### Supplementary Information

**Additional file 1: Figure S1.** Production of T_RM_ cells in Study 1 with FTY720 treatment. Mice were orally administered FTY720 7 days before intranasal boost immunization, lasting for 30 days after intranasal boost immunization until the time to determine lung T_RM_ cells, including CD4^+^CD69^+^ (A), CD4^+^CD69^+^CD103^+^ (B), CD4^+^CD69^+^CD103^−^ (C), CD8^+^CD69^+^ (D), CD8^+^CD69^+^CD103^+^ (E) and CD8^+^CD69^+^CD103^−^ (F) subtypes (n = 4).

**Additional file 2: Figure S2.** Production of T_RM_ cells in Study 2 with FTY720 treatment. Mice were orally administered FTY720 7 days before intranasal boost immunization, lasting for 30 days after intranasal boost immunization until the time to determine lung T_RM_ cells, including CD4^+^CD69^+^ (A), CD4^+^CD69^+^CD103^+^ (B), CD4^+^CD69^+^CD103^−^ (C), CD8^+^CD69^+^ (D), CD8^+^CD69^+^CD103^+^ (E) and CD8^+^CD69^+^CD103^−^ (F) subtypes (n = 4, *P < 0.05, ns, not significant).

**Additional file 3: Figure S3.** Demonstration of the deduced principle of DC-targeting vaccine-generated lung T_RM_.

**Additional file 4: Figure S4.** Gating strategy for flow cytometer analysis of effector memory T cells in spleen.

**Additional file 5: Figure S5.** Gating strategy for flow cytometer analysis of lung resident memory T cells.

**Additional file 6: Figure S6.** Gating strategy for flow cytometer analysis of CD11b^+^ and CD103^+^ lung dendritic cells.

**Additional file 7: Figure S7.** Gating strategy for flow cytometer analysis of production of intracellular cytokines in lung.

**Additional file 8: Figure S8.** Gating strategy for flow cytometer analysis of production of intracellular cytokines in spleen.

## Data Availability

All data generated or analysed during this study are included in this published article.
