# Supplementary figures and images for: A novel “prime and pull” strategy mediated by the combination of two dendritic cell-targeting designs induced protective lung tissue-resident memory T cells against H1N1 influenza virus challenge
Source: J Nanobiotechnology. 2023 Dec 13;21:479. doi: 10.1186/s12951-023-02229-y (PMC10717309; doi:10.1186/s12951-023-02229-y)

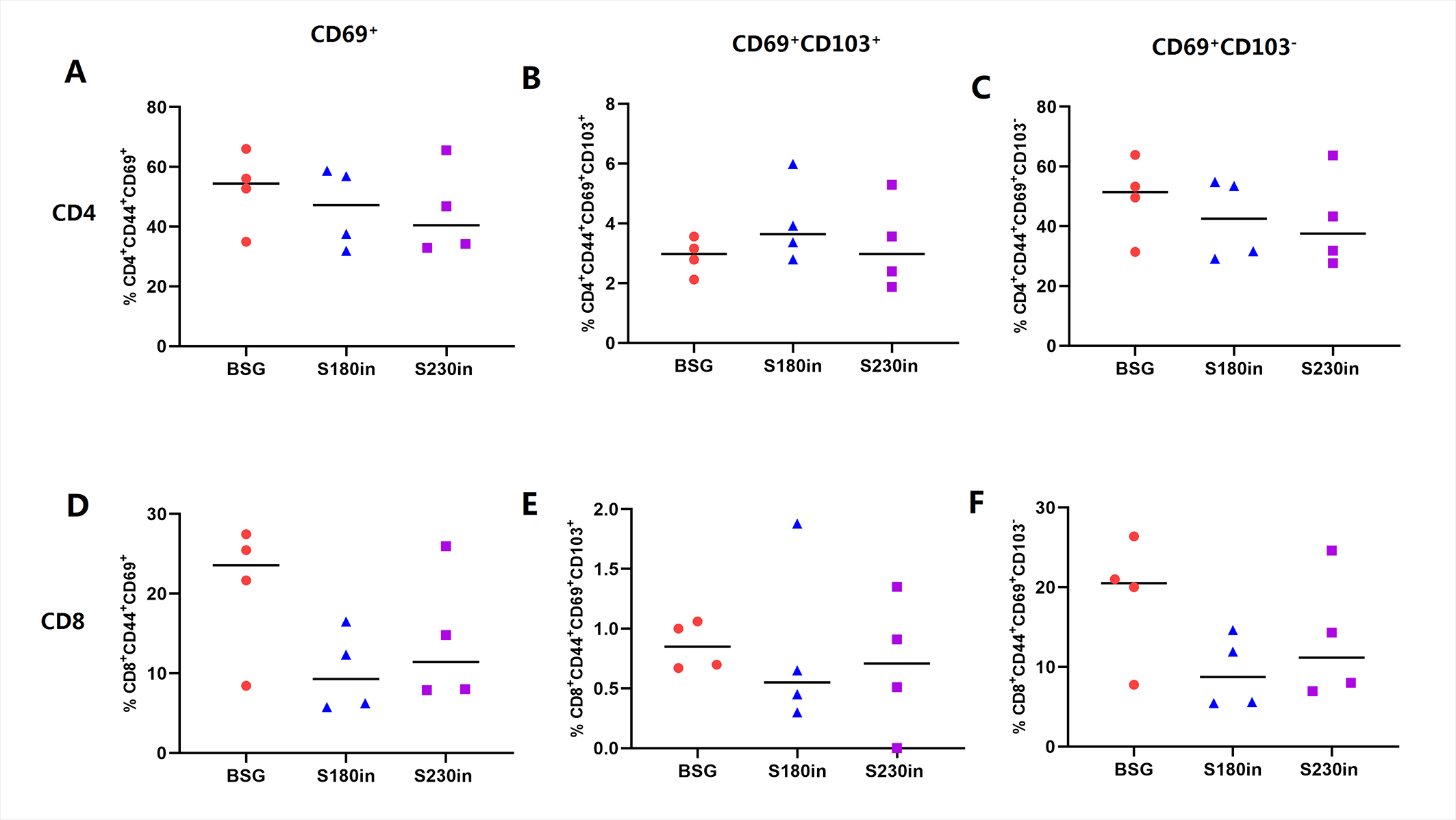

Supplement: Supplementary file 1 — Additional file 1: Figure S1. Production of TRM cells in Study 1 with FTY720 treatment. Mice were orally administered FTY720 7 days before intranasal boost immunization, lasting for 30 days after intranasal boost immunization until the time to determine lung TRM cells, including CD4+CD69+ (A), CD4+CD69+CD103+ (B), CD4+CD69+CD103− (C), CD8+CD69+ (D), CD8+CD69+CD103+ (E) and CD8+CD69+CD103− (F) subtypes (n = 4). [file 12951_2023_2229_MOESM1_ESM.tif]

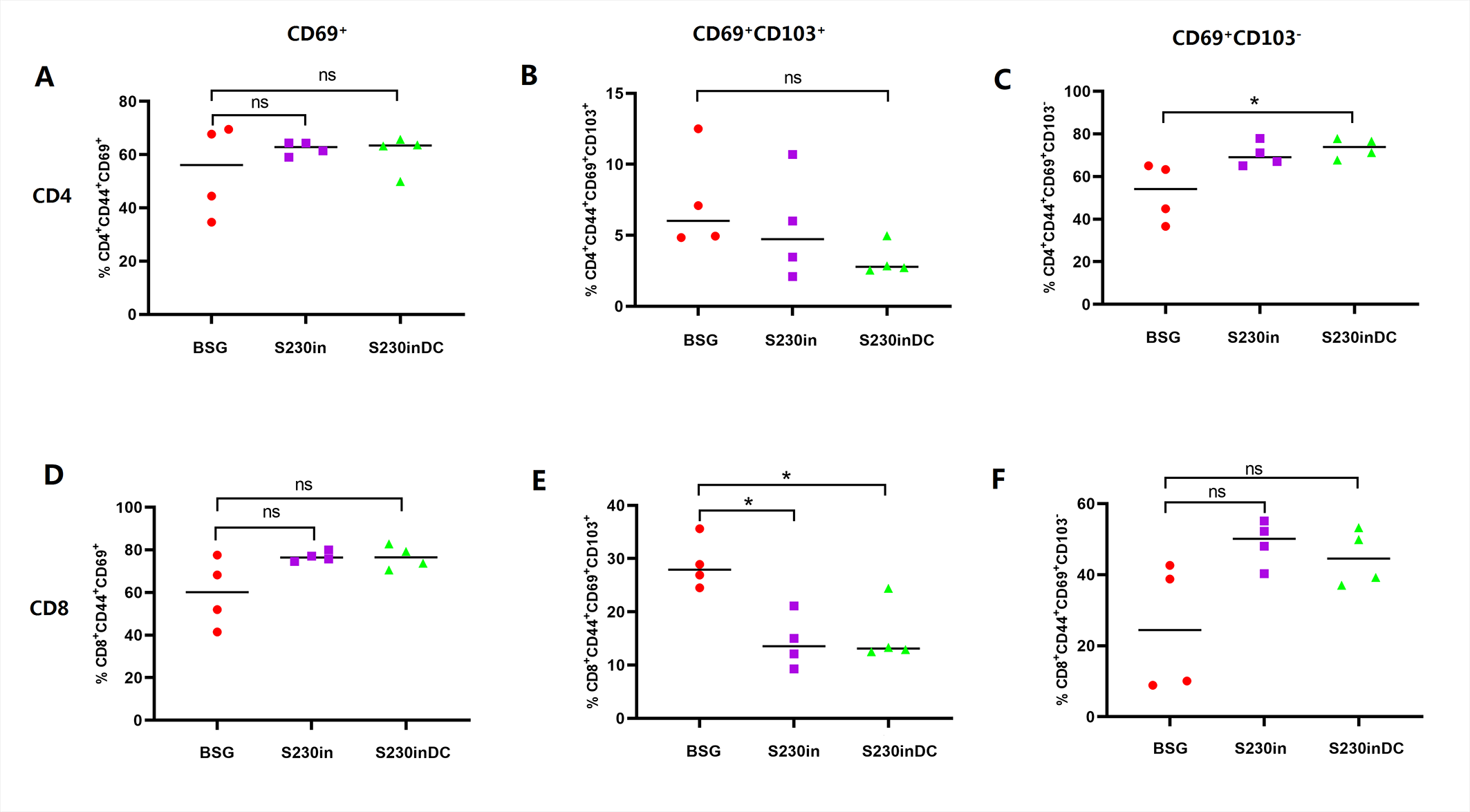

Supplement: Supplementary file 2 — Additional file 2: Figure S2. Production of TRM cells in Study 2 with FTY720 treatment. Mice were orally administered FTY720 7 days before intranasal boost immunization, lasting for 30 days after intranasal boost immunization until the time to determine lung TRM cells, including CD4+CD69+ (A), CD4+CD69+CD103+ (B), CD4+CD69+CD103− (C), CD8+CD69+ (D), CD8+CD69+CD103+ (E) and CD8+CD69+CD103− (F) subtypes (n = 4, *P < 0.05, ns, not significant). [file 12951_2023_2229_MOESM2_ESM.tif]

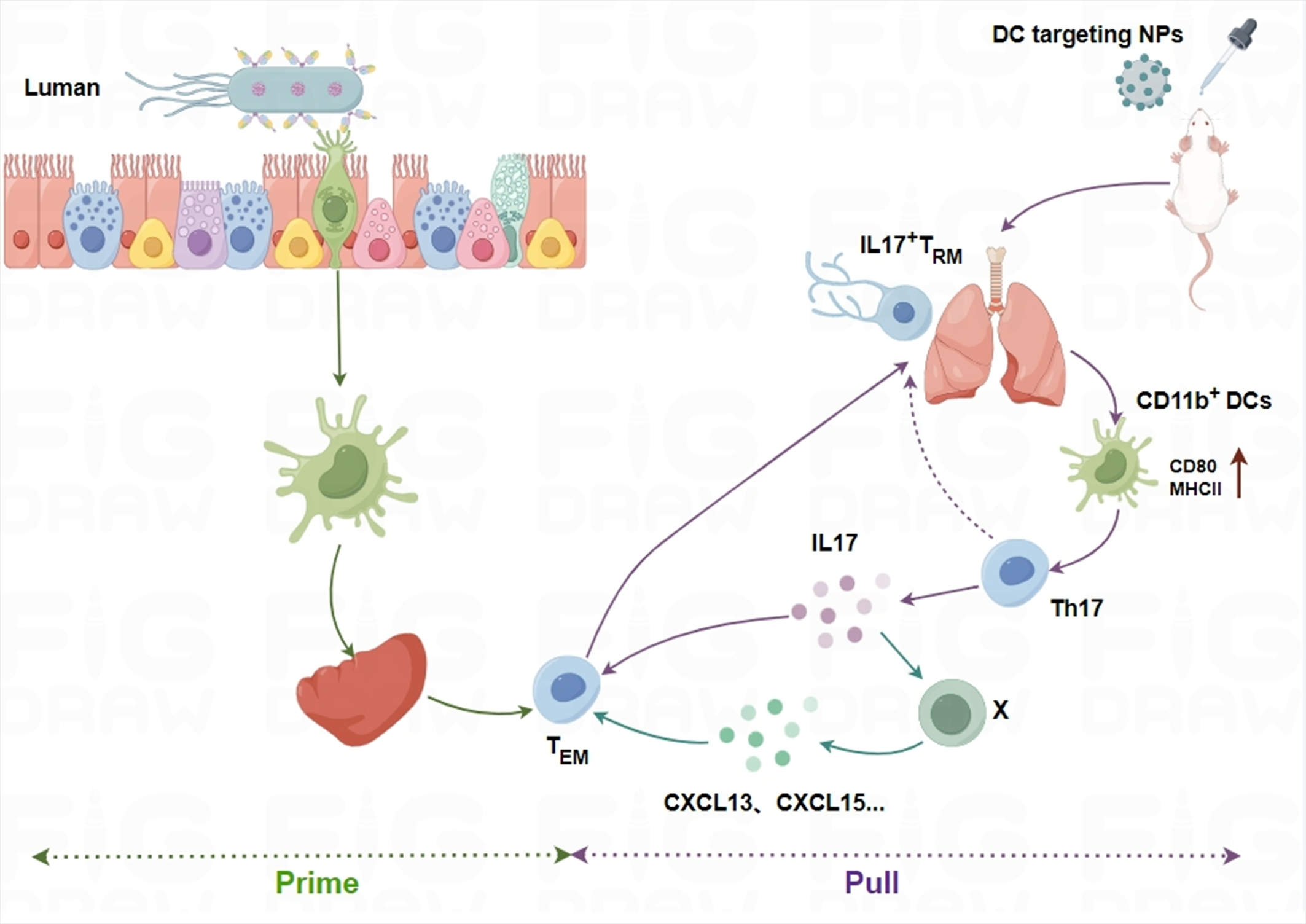

Supplement: Supplementary file 3 — Additional file 3: Figure S3. Demonstration of the deduced principle of DC-targeting vaccine-generated lung TRM. [file 12951_2023_2229_MOESM3_ESM.tif]

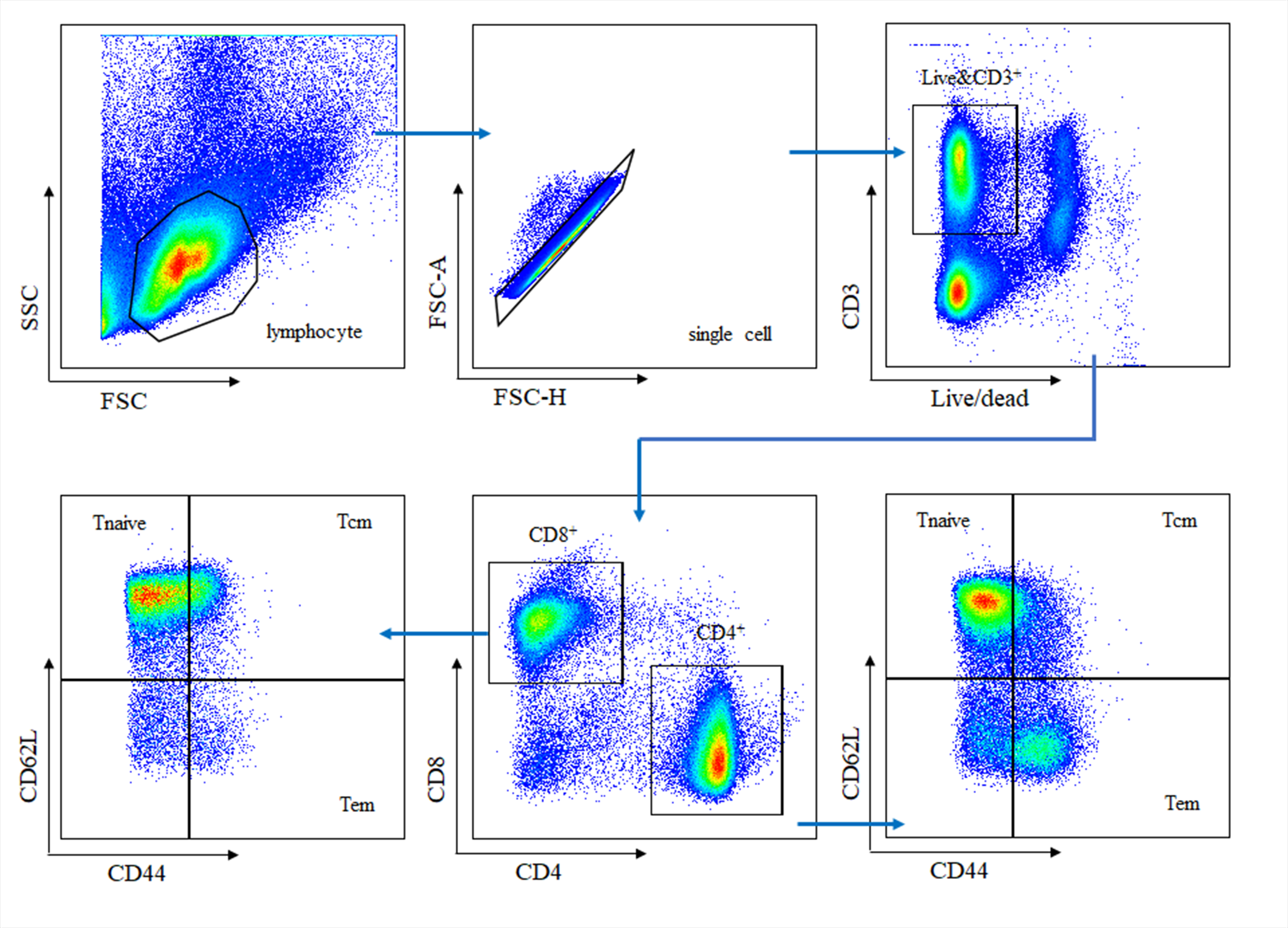

Supplement: Supplementary file 4 — Additional file 4: Figure S4. Gating strategy for flow cytometer analysis of effector memory T cells in spleen. [file 12951_2023_2229_MOESM4_ESM.tif]

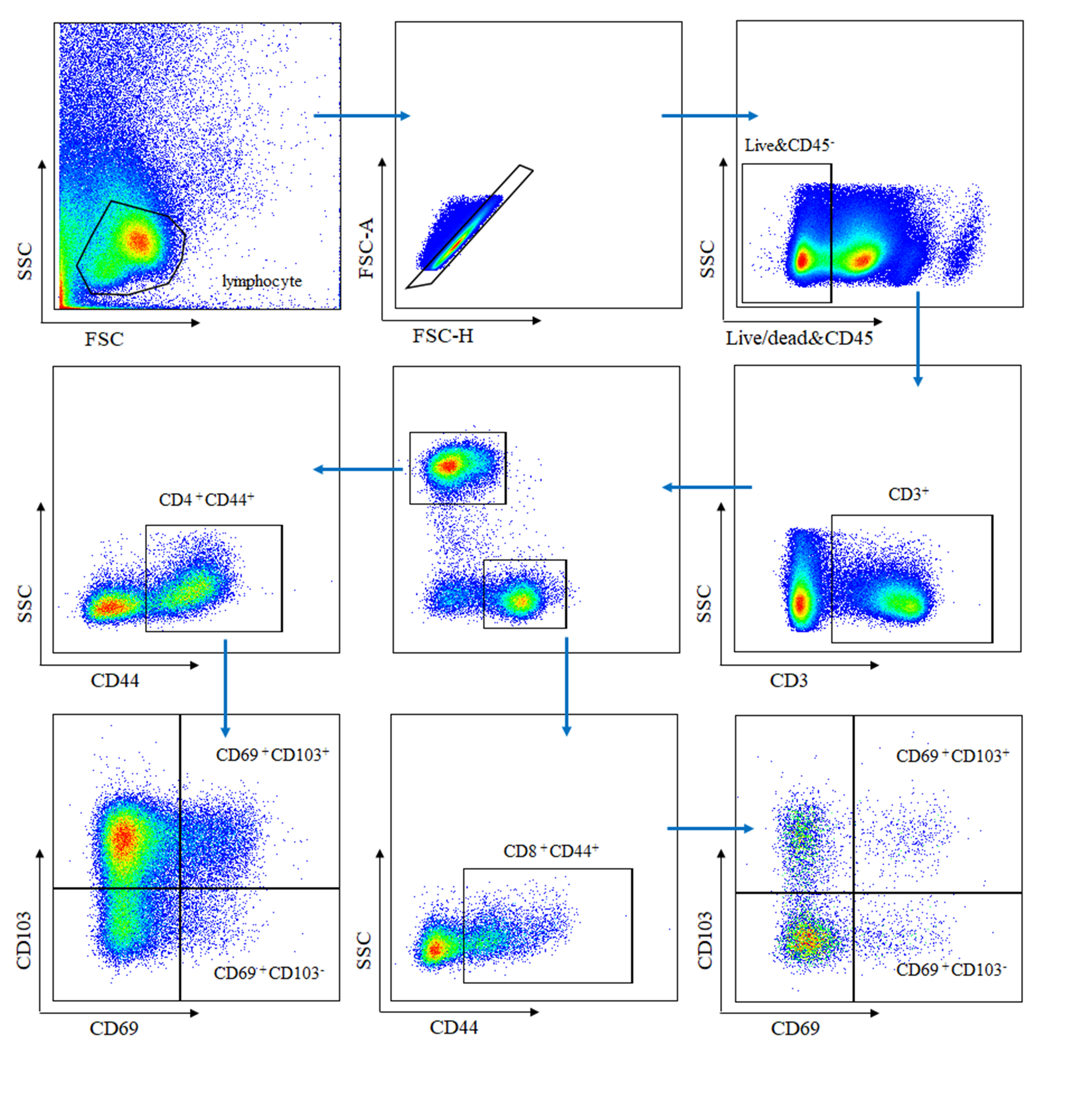

Supplement: Supplementary file 5 — Additional file 5: Figure S5. Gating strategy for flow cytometer analysis of lung resident memory T cells. [file 12951_2023_2229_MOESM5_ESM.tif]

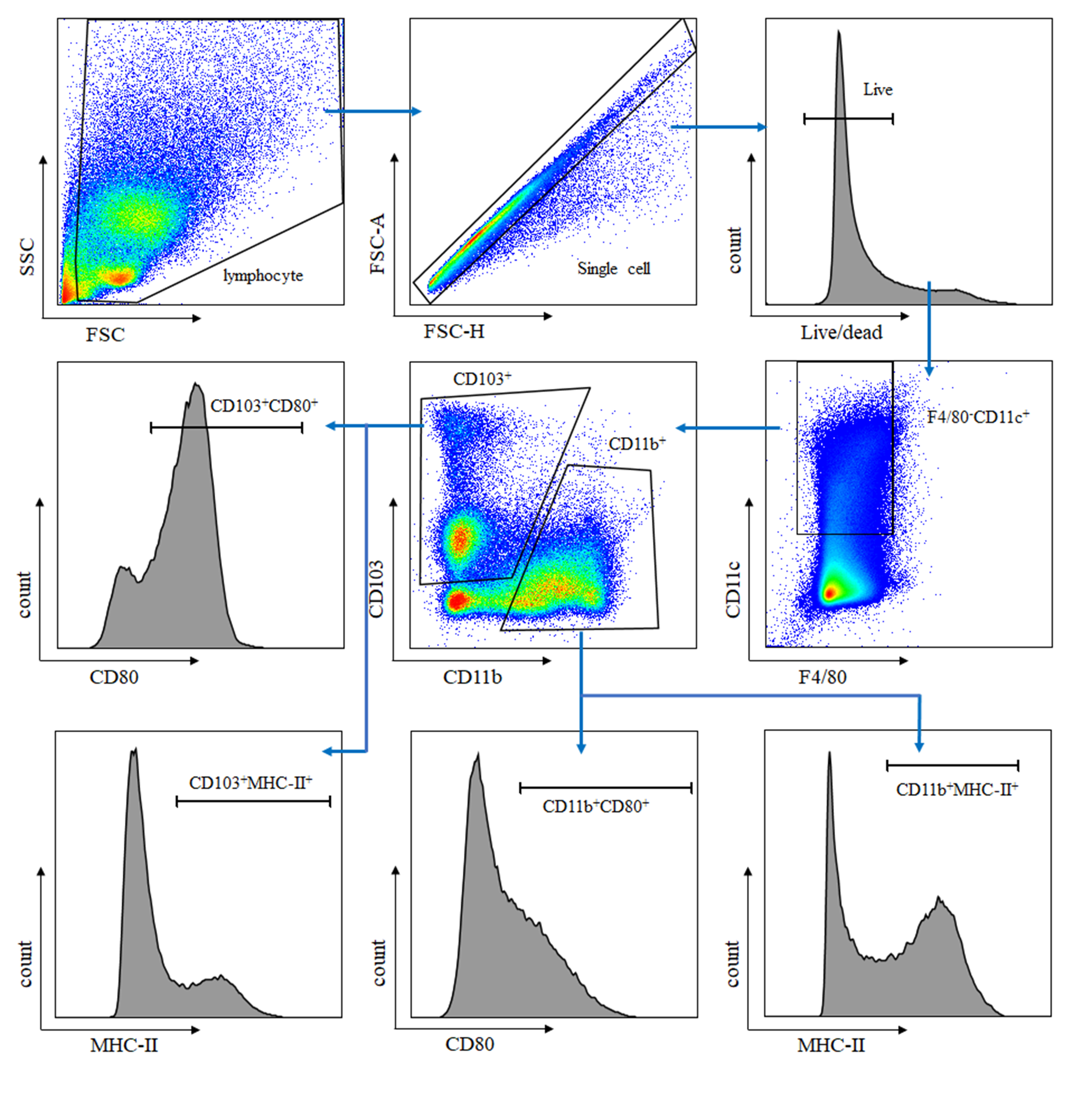

Supplement: Supplementary file 6 — Additional file 6: Figure S6. Gating strategy for flow cytometer analysis of CD11b+ and CD103+ lung dendritic cells. [file 12951_2023_2229_MOESM6_ESM.tif]

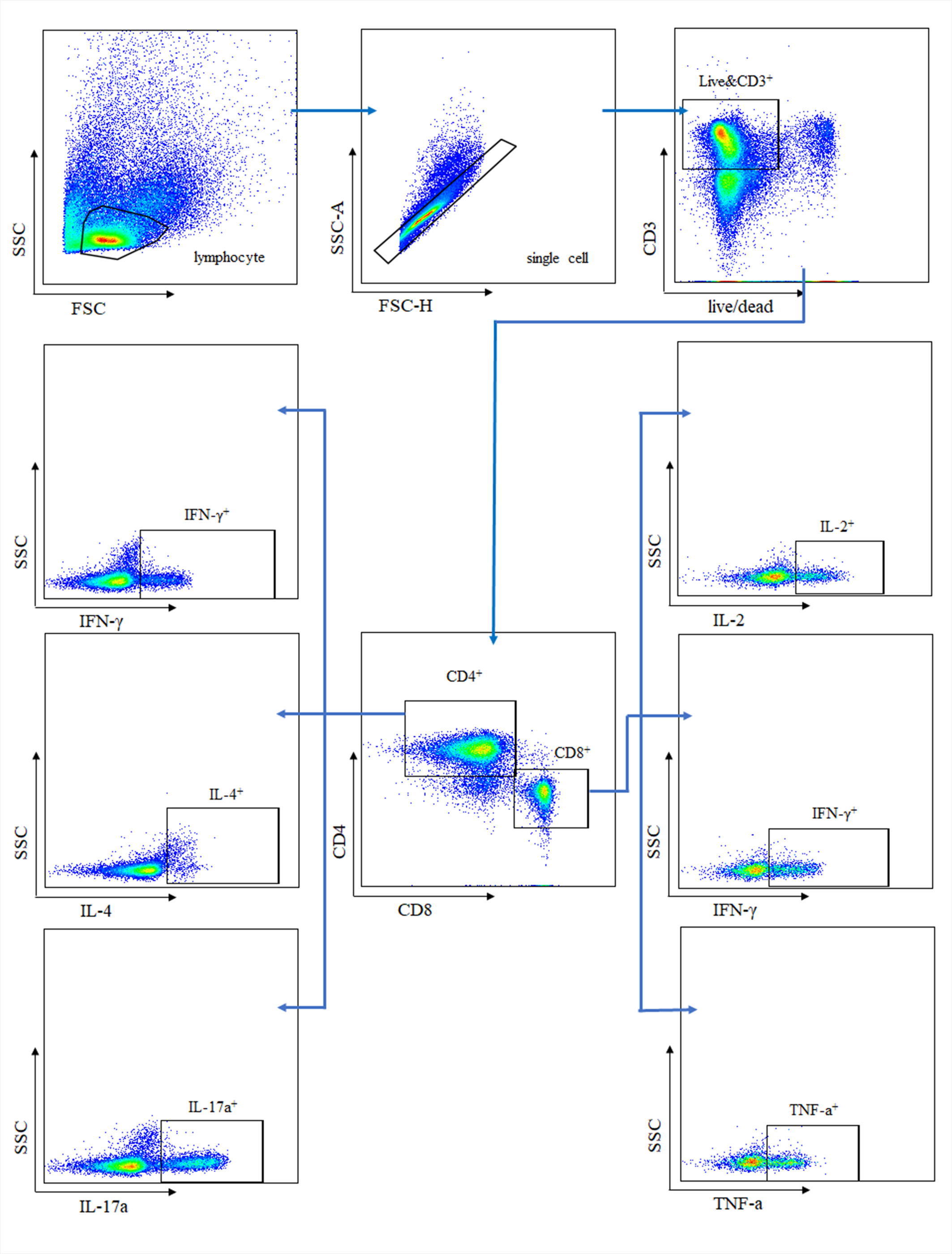

Supplement: Supplementary file 7 — Additional file 7: Figure S7. Gating strategy for flow cytometer analysis of production of intracellular cytokines in lung. [file 12951_2023_2229_MOESM7_ESM.tif]

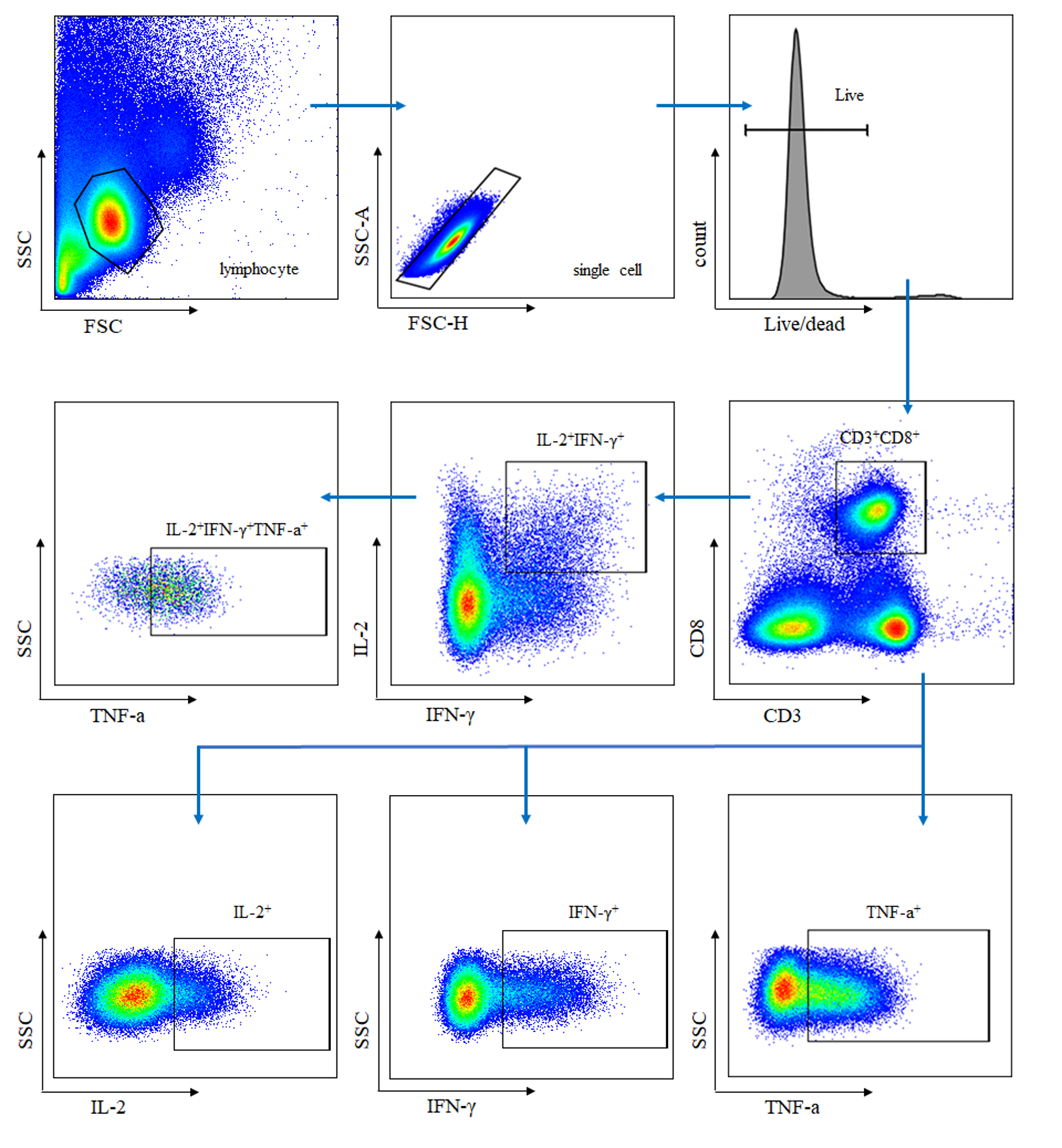

Supplement: Supplementary file 8 — Additional file 8: Figure S8. Gating strategy for flow cytometer analysis of production of intracellular cytokines in spleen. [file 12951_2023_2229_MOESM8_ESM.tif]
